# Privacy by Projection: Federated Population Density Estimation by Projecting on Random Features

**DOI:** 10.56553/popets-2023-0019

**Published:** 2023-07

**Authors:** Zixiao Zong, Mengwei Yang, Justin Ley, Carter T. Butts, Athina Markopoulou

**Affiliations:** University of California, Irvine, Irvine, CA, USA; University of California, Irvine, Irvine, CA, USA; University of California, Irvine, Irvine, CA, USA; University of California, Irvine, Irvine, CA, USA; University of California, Irvine, Irvine, CA, USA

**Keywords:** Kernel Density Estimation (KDE), Privacy, Random Fourier Features, Federated Analytics, Population Modeling

## Abstract

We consider the problem of population density estimation based on location data crowdsourced from mobile devices, using kernel density estimation (KDE). In a conventional, centralized setting, KDE requires mobile users to upload their location data to a server, thus raising privacy concerns. Here, we propose a Federated KDE framework for estimating the user population density, which not only keeps location data on the devices but also provides probabilistic privacy guarantees against a *malicious* server that tries to infer users’ location. Our approach Federated random Fourier feature (RFF) KDE leverages a random feature representation of the KDE solution, in which each user’s information is irreversibly projected onto a small number of spatially delocalized basis functions, making precise localization impossible while still allowing population density estimation. We evaluate our method on both synthetic and real-world datasets, and we show that it achieves a better utility (estimation performance)-vs-privacy (distance between inferred and true locations) tradeoff, compared to state-of-the-art baselines (e.g., GeoInd). We also vary the number of basis functions per user, to further improve the privacy-utility trade-off, and we provide analytical bounds on localization as a function of areal unit size and kernel bandwidth.

## 1 INTRODUCTION

With the widespread use of smart phones and wearable devices in recent years, location data has become increasingly available. This has enabled several modeling tasks, including population density [43], which is the focus of this paper. One application is to provide a data-driven perspective for public transit operators, since it can capture customer mobility patterns and inform resource allocation in urban areas [6, 21, 29, 33]. In addition, the relationship between population density and infectious disease is of considerable public health relevance [5, 17, 32], especially during the COVID-19 pandemic [3, 23, 35, 48]. Local differences in population density and interaction rates can have substantial impacts on the community risk levels [41, 42], but information about people’s locations and movements is clearly sensitive.

In some cases, user location data is publicly available from administrative or social media sources [50, 51] or contributed by survey participants [14]. Other cases involve the geospatial information crowdsourced from users employing location-aware apps such as Google maps and Waze, which frequently track users’ movements in fine detail and without supervision. The collection of data from such apps raises privacy concerns [22]. Potential disclosure of geolocation data has negative impacts on both users and prospective analysts: not only may disclosure directly harm users, but measures taken by users (or developers, on users’ behalf) to avoid such disclosures may inhibit sharing of useful information that could improve apps’ performance, allow new services to be offered, etc. There is thus considerable interest in privacy-preserving approaches to the collection and analysis of crowdsourced geospatial data.

In this paper, we focus on the problem of modeling population density from individual geolocations, collected e.g. from mobile applications. Population density is estimated on a grid with a chosen range and interval, where density at each coordinate is estimated from users’ locations. The estimated density surface may then be used for visualization, or as an input to other analysis or prediction tasks. Our objective is to perform this task in a distributed manner, in such a way that users do not share their location with the server, and such information cannot be inferred by a malicious server.

More specifically, we consider a federated framework, which is today’s preeminent paradigm for distributed learning (FL) and analytics (FA). User data is stored and processed locally on the devices, and only the result of a local computation (e.g., the model updates in FL) are sent from the devices to the server [24, 31, 44]. In the context of federated population density estimation, this enables the server to estimate the population distribution, by aggregating all users’ updates, while individual users can still keep the raw geolocation data on their devices. For the purpose of estimating population distributions, Kernel Density Estimation (KDE) [10] is a natural fit: it is non-parametric, computationally, and naturally lends itself to a federated implementation.

Unfortunately, even in a Federated KDE setting where users’ data is not directly disclosed, a malicious server can still infer users’ locations, by querying users for local density information and using it to deduce their most probable locations. A range of privacy-preserving techniques have been developed and added onto the basic federated learning and analytics frameworks, including differential privacy (DP) on the mobiles and/or the server [16, 27, 34], secure aggregation [7, 15], and combinations thereof [25], [3].

In this paper, we propose a new privacy-preserving technique for Federated KDE, orthogonal to existing defenses, to help protect users against location inference by a malicious server, which we refer to as *Federated RFF KDE*. The idea is to *project user data onto a small number of spatially delocalized functions*^1^ *in Fourier space* - from which user’s location cannot be inferred - and perform KDE using a version of the random feature method [39] (RFF). Using both synthetic and real-world data, we show empirically that the proposed method is able to achieve excellent approximations to the density surface with even a few random features, under realistic conditions. Moreover, we also show that this is sufficient to prevent users from being localized. These studies are complemented by theoretical analysis proving that user locations cannot be inferred from the spatially delocalized projections, making it impossible for a malicious server to localize a user, even given an unlimited number of queries.

**Figure 1:**
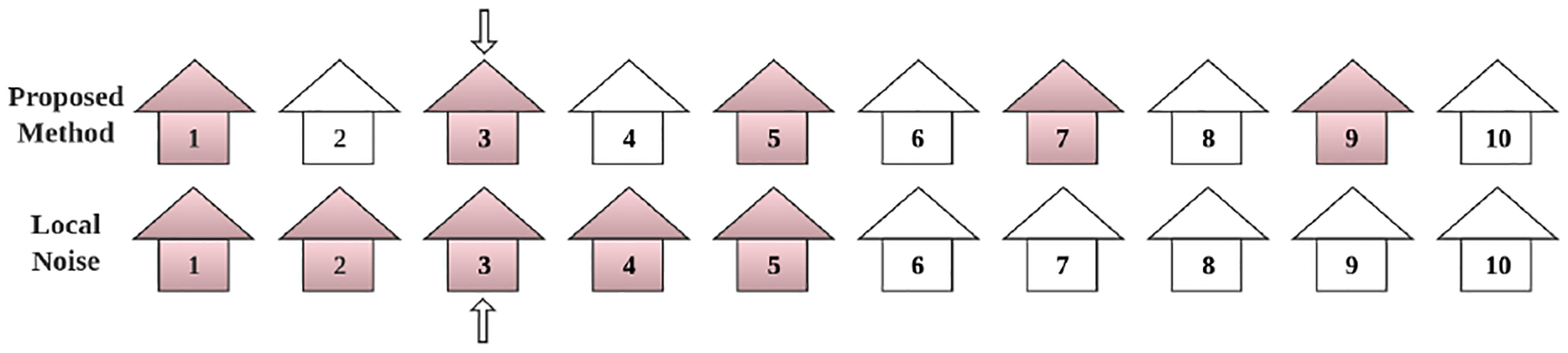
Discrete 1D example of a feature projection. Assume there are 10 houses along a street, participating in FL, and the server queries the user residing in house 3 for a single (one-time) local update. A scheme adding (spatially local) noise might randomly perturb the user’s location, putting them in any of the houses from 1 to 5, all of which are relatively close to the user’s true location, and will reveal the part of the street on which the user lives (e.g., left or right half). Our projection method is akin to revealing whether the user lives in an even or odd house. Although the same number of houses (5) are excluded, the remaining set is distributed over the entire length of the street, thus hiding the user’s relative position. In that sense, the even and odd features, and corresponding projections onto them, as “spatially delocalized”.

Next, we explain the intuition of our proposed method and compare it to that of local noise-adding (including DP-based) privacy-preserving approaches for location-based applications [27, 34]. To protect against a malicious server, most existing techniques conceal users’ locations by adding local noise (on the data and/or updates). However, such obfuscation is still relatively *localized*, since the user’s true location will still be close to the noised version, with their expected distance depending on the noise added. Our proposed method is completely spatially delocalized. Specifically, the proposed method does not add any noise to data but abstracts information from users’ data by nonlinear projection to a set of basis functions whose symmetry group does not allow a user’s data to be distinguished from other data in an equivalence class that is distributed over the entire plane. Rather than trying to hide the user by obfuscating their data, then, we reveal only the equivalence class to which it belongs - a class containing a potentially infinite number of other datasets, spread out through space. Fig. 1 attempts to illustrate the intuition of how our method works, using a discrete, one-dimensional example of a feature projection. Although our function space is richer than the simple example of Fig. 1, the intuition generalizes: we selectively remove information in a way that efficiently protects location over the whole map, rather than adding local noise (which obfuscates local location but does not efficiently conceal global location).

The remainder of the paper is organized as follows. Section 2 details the problem setting and notation, and provides brief overviews of Kernel Density Estimation and Random Fourier Features. In section 3, we demonstrate how our proposed method works to estimate density and protect privacy, and we provide the theoretical analysis regarding bandwidth restriction and privacy preservation. In section 4, we present numerical experiments on both synthetic and real-world data to show the effectiveness of the proposed method, and compare its privacy and performance with baseline methods and alternatives. Section 5 discusses related work about federated learning privacy protection schemes and location privacy, and section 6 concludes the paper.

## 2 PRELIMINARIES

### 2.1 Notation and Problem Setting

Although our approach can be used for any density estimation problem, for concreteness we focus on a setting in which we have N users, each of whom is associated with a location ${\text{d}}_{i}$ (We treat ${\text{d}}_{i}$ as a two-dimensional coordinate vector, although the majority of our results generalize immediately to 1D or to higher dimensions). In our setting, we assume that this location information is privately held by the users, and is only available to the analyst (server) when explicitly shared. For notational convenience, however, we denote the full dataset by $\text{D}=\left\{{\text{d}}_{1},{\text{d}}_{2},\;\ldots \;,{\text{d}}_{N}\right\}$. Our problem is for a central server to reconstruct the population density associated with D, without having direct access to any location ${\text{d}}_{i}$. Moreover, we wish to prevent a malicious server from being able to *infer* user locations by repeated querying.

Without loss of generality, we consider our problem on a rectangular region A (Any non-rectangular region can be generalized to a rectangular region via its bounding box). As a practical matter, we treat the target density via levels on a P×Q grid in this area, with $G\left({\text{g}}_{pq}\right)$ being the density obtained at location (p,q) estimated over all N. data points. Our focus is on obtaining density estimates that approximate $G\left({\text{g}}_{pq}\right)$ and that can be calculated without direct access to the elements of D (and without allowing elements of D to be inferred). Throughout, density estimation is performed via kernel density estimation, as described below.

### 2.2 Kernel Density Estimation

Kernel density estimation (KDE) is a non-parametric method to estimate a density function from a set of random draws from the corresponding distribution [37]. In our setting, assuming the underlying density function $f_{X}$, the kernel density estimator of $f_{X}$ at x is:

(1) $$f\left(\text{x}|\text{D}\right)=\frac{1}{N}\overset{N}{\underset{i=1}{\sum }}k_{h}\left(\text{x},{\text{d}}_{i}\right)$$

where x is any vector in A, and $k_{h}$ is any kernel *function* with bandwidth h. In this context, a kernel function is a symmetric, non-negative function with a unit integral over the space of x. A common and flexible choice of k is the Gaussian kernel, $k_{h}\left(x,y\right)\equiv \text{exp}\left(-\frac{\Delta^{2}}{2h^{2}}\right)$, where $\Delta =∥x-y{∥}_{2}^{2}$. This choice is especially convenient for our privacy-preserving algorithm, and we use it throughout, though generalization to other shift-invariant kernels (i.e., it satisfies k(x,y)=k(x−y,0)) is possible.

### 2.3 Random Fourier Features

Since the Gaussian kernel is shift-invariant, it follows from Bochner’s theorem [39] that,

(2) $$k\left(\text{x},\text{y}\right)=\int p\left(\omega \right)e^{j\omega^{⊤}\left(\text{x}-\text{y}\right)}d\omega =E\left[e^{j\omega^{⊤}\left(\text{x}-\text{y}\right)}\right]$$

where p(ω) is kernel k’s corresponding Fourier density. This means that one is able to use Monte-Carlo sampling to achieve the expectation in (2) with

(3) $$E\left[e^{j\omega^{⊤}\left(\text{x}-\text{y}\right)}\right]\approx \frac{1}{B}\overset{B}{\underset{b=1}{\sum }}e^{j\omega_{b}^{⊤}\left(\text{x}-\text{y}\right)}\;=\frac{1}{B}\overset{B}{\underset{b=1}{\sum }}\phi_{b}\left(\text{x},\text{y}\right)=\frac{1}{B}\text{z}{\left(\text{x}\right)}^{⊤}\text{z}\left(\text{y}\right)$$

where $\phi_{b}$ is a randomly chosen function from the Fourier basis of k and B is the number of sampled functions. The basis functions have the form

(4) $$\phi_{b}\left(\text{x},\text{y}\right)=\left[\text{cos}\left(\omega_{b}^{⊤}\text{x}\right),\text{sin}\left(\omega_{b}^{⊤}\text{x}\right)\right]{\left[\text{cos}\left(\omega_{b}^{⊤}\text{y}\right),\text{sin}\left(\omega_{b}^{⊤}\text{y}\right)\right]}^{⊤}$$

where $\omega_{b}$ is a random vector sampled i.i.d from the Fourier density p(ω). Selecting B such functions then gives us the *random feature* matrices

$$\text{z}\left(\text{x}\right)={\left[\text{cos}\left(\omega_{1}^{⊤}\text{x}\right),\text{sin}\left(\omega_{1}^{⊤}\text{x}\right),\;\cdots \;,\text{cos}\left(\omega_{B}^{⊤}\text{x}\right),\text{sin}\left(\omega_{B}^{⊤}\text{x}\right)\right]}^{⊤}$$

which are the projections of the original data onto the randomly chosen basis functions. For the Gaussian kernel, the random vector ω with bandwidth h is sampled from $𝒩\left(0,h^{-2}I\right)$. We use this kernel and random feature representation in our subsequent development.

A property of the Fourier basis that is important for our application is that the basis functions are *spatially delocalized*: they are sinusoidal functions that span the entire input space, and are moreover invariant to translations orthogonal to their “direction of motion” (as well as translations of integer multiples of their wavelength along their direction of motion). Individually, such features contain very little spatial information. This will be of use in building a privacy-preserving federated KDE algorithm.


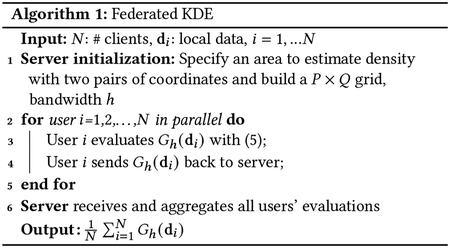


## 3 METHODS

### 3.1 Baseline: Federated KDE

Because the kernel density estimator is linearly separable over the data, KDE naturally lends itself to federation. The idea is that users’ location data ${\text{d}}_{i}$ can be kept local but for a specific coordinate ${\text{g}}_{pq}$, the work of evaluating $f\left({\text{g}}_{pq}|\text{D}\right)$ can be distributed across users. Each user i evaluates $f\left({\text{g}}_{pq}|{\text{d}}_{i}\right)$, and then sends it back to the server. The server receives all users’ evaluations and obtains the density estimate at ${\text{g}}_{pq}$ by averaging over $f\left({\text{g}}_{pq}|{\text{d}}_{i}\right)$.

More specifically, the server starts by specifying an area A and defining a grid with the desired resolution. It shares the coordinates P×Q of the grid and the kernel bandwidth h with all users. The server asks all users in this area to evaluate the kernel function values at all coordinates on the grid with a specified bandwidth. Each user evaluates kernel function values at all coordinates on the grid and sends them back to the server. The server averages all users’ evaluations to derive the overall density surface over the target area A. Formally speaking, a user i will evaluate $G_{h}\left({\text{d}}_{i}\right)$ along the P×Q grid with bandwidth h, producing

(5) $$G_{h}\left({\text{d}}_{i}\right)=\left(\begin{array}{ccc} k_{h}\left({\text{d}}_{i},{\text{g}}_{11}\right) & \cdots & k_{h}\left({\text{d}}_{i},{\text{g}}_{1Q}\right)\\ \vdots & \ddots & \vdots \\ k_{h}\left({\text{d}}_{i},{\text{g}}_{P1}\right) & \cdots & k_{h}\left({\text{d}}_{i},{\text{g}}_{PQ}\right) \end{array}\right)$$

where ${\text{g}}_{pq}$ is the coordinate on the grid. By receiving $G_{h}\left({\text{d}}_{i}\right)$ from all users, the overall density surface can be estimated by aggregating $\frac{1}{N}\sum_{i=1}^{N}G_{h}\left({\text{d}}_{i}\right)\in {\mathbb{R}}^{P\times Q}$. This procedure is shown in Alg. 1.

Note that as previously stated, the 2D location problem is for concreteness, but the proposed framework can be utilized in any dimension. In a 1D scenario, both g and d are in ℝ, and $G_{h}\left(\text{d}\right)$ will be a vector instead of a matrix. In 3D cases, such as spatial-temporal data, $G_{h}\left(\text{d}\right)$ will be a 3D tensor. Higher dimensions are possible, although as a practical matter KDE is usually used in low-dimensional settings.

#### Privacy Attack.

**Figure 2:**
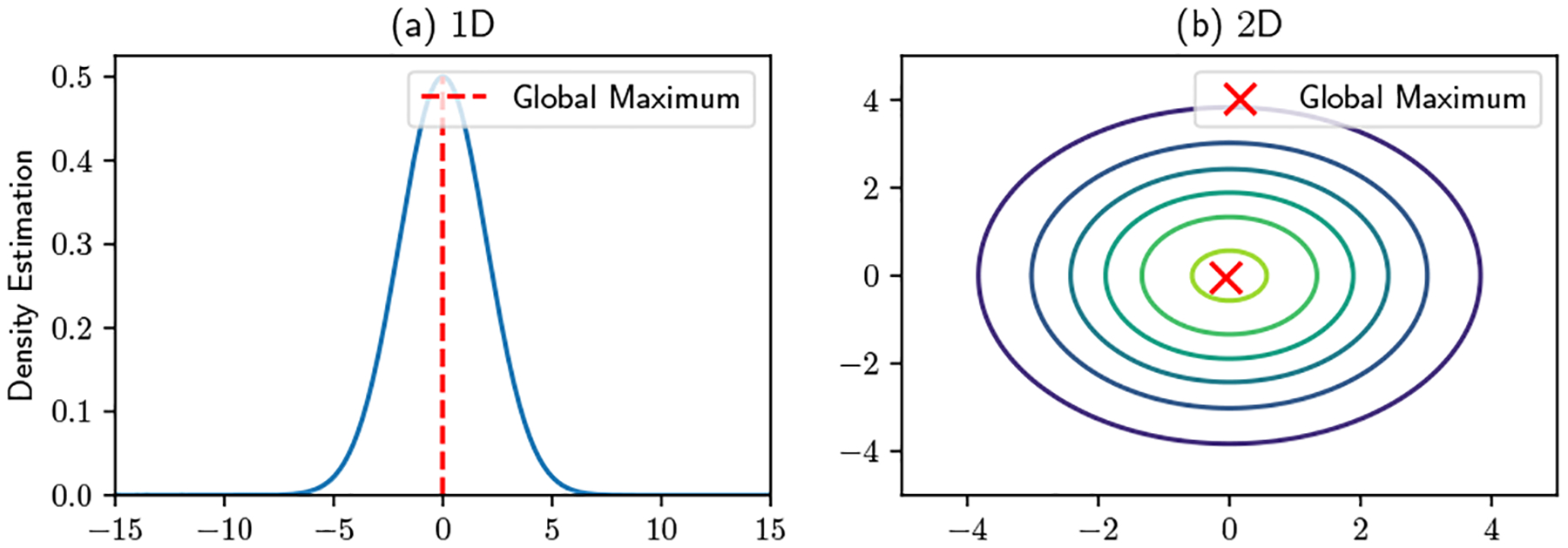
Privacy attack in Federated KDE: the server can infer a user’s location to be close to the coordinates of maximum evaluation.

Even though Federated KDE does not directly share users’ data with the server, a user’s function evaluation(s) may reveal to the server that user’s location information. To illustrate the intuition of this inference attack, Fig. 2 shows 1D and 2D examples of a single user’s local evaluations, which are visualizations of what a user actually sends back to the server. In Fig. 2 (a) the data is at 0, and the kernel function is evaluated at 1000 points which are evenly spaced over the interval [−15, 15]. With an arbitrary bandwidth 2, one can observe that the coordinate of maximum evaluation is close to 0. Similarly, in Fig 2 (b) the user’s location is (0, 0), and the kernel function is evaluated on a 100×100 grid over [−5, 5] × [−5, 5] Again, the location of the global maximum is close to (0, 0). The proximity of the global maximum to the user’s location is only limited by the resolution of the grid. Assuming that the server can specify a sufficiently dense grid, it can infer each user’s location to arbitrary precision. This provides the intuition behind location inference in the Federated KDE setting, while the full description of the adversary is provided in section 3.3.

### 3.2 Proposed Algorithm: Federated RFF KDE

To protect against the aforementioned privacy attack, we propose an improved method that, instead of using the exact kernel function $k_{h}$, it approximates the kernel with the random Fourier features (RFF) of section 2.3. In particular, instead of providing kernel evaluations, each user calculates and returns an approximation obtained by projecting their data onto a small number of random features (possibly only one). Substituting (3) into the definition of kernel density estimation (1), we can express our approximation $f^{\prime }$ as an estimator of f as

(6) $$f\left(\text{x}|\text{D}\right)\approx f^{\prime }\left(\text{x}|\text{D}\right)=\frac{1}{NB}\overset{N}{\underset{i=1}{\sum }}\overset{B}{\underset{b=1}{\sum }}\phi_{b}^{i}\left(\text{x},{\text{d}}_{i}\right)$$

where $f^{\prime }$ is the random feature based kernel density estimator, and $\phi_{b}^{i}$ is user i’s bth basis function. Then, instead of every user i evaluating kernel function $k_{h}\left({\text{g}}_{pq},{\text{d}}_{i}\right)$ at each coordinate on the grid, B basis functions are used to approximate each user’s evaluations. As with Federated KDE in Alg. 1, (6) can also be implemented via federated learning. The proposed algorithm, Federated RFF KDE, is shown as Alg. 2 and described next.

First, the server specifies a grid over the area to be estimated. Next, all users inside the area are queried for function evaluations at each grid point using a specific number of random features B, and a bandwidth h. In response, each user samples B random basis functions with bandwidth h as requested, and returns the projection of their location data onto these functions, evaluated at the selected grid pairs. Note that all sampled random vectors are stored locally, and are not accessible to either the server or to other users. (In “one shot” applications, the vectors may further be discarded, making them inaccessible even to one who subsequently gains access to the user’s device.) Moreover, since both generations of random vectors and query response are handled locally, the user can refuse “improper” queries from the server (e.g., requests to evaluate at more than B basis functions). Importantly, in the multiple-query cases, a user does not re-draw random vectors between responses. This ensures that nothing can be learned beyond its spatially delocalized projections. An important special case of the latter is when the server issues queries with multiple bandwidth choices (as may occur if tuning is performed). Instead of drawing a new ω, the user only samples ω from $𝒩\left(0,h_{0}^{-2}\text{I}\right)$ once, and when a server’s query with bandwidth h comes, the user rescales the sampled value with $\frac{h_{0}}{h}\omega$, where $h_{0}$ is user’s previous bandwidth. How this preserves privacy is further discussed in section 3.5.


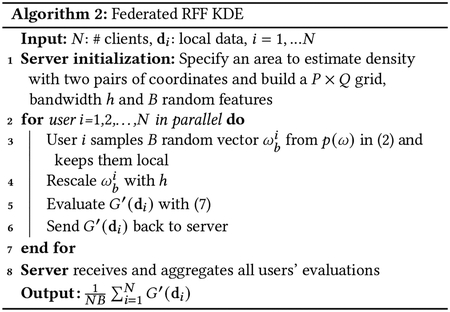


After drawing B basis functions, user i’s local evaluations can then be expressed as

(7) $$G^{\prime }\left({\text{d}}_{i}\right)=\left(\begin{array}{ccc} \sum_{b=1}^{B}\phi_{b}^{i}\left({\text{d}}_{i},{\text{g}}_{11}\right) & \cdots & \sum_{b=1}^{B}\phi_{b}^{i}\left({\text{d}}_{i},{\text{g}}_{1Q}\right)\\ \vdots & \ddots & \vdots \\ \sum_{b=1}^{B}\phi_{b}^{i}\left({\text{d}}_{i},{\text{g}}_{P1}\right) & \cdots & \sum_{b=1}^{B}\phi_{b}^{i}\left({\text{d}}_{i},{\text{g}}_{PQ}\right) \end{array}\right)$$

where $\phi_{b}^{i}$ is corresponding to user i’s random vector $\omega_{b}^{i}$ as (4). The server collects each user’s $G^{\prime }\left({\text{d}}_{i}\right)$ and adds them, obtaining the estimated density surface with $\frac{1}{NB}\sum_{i=1}^{N}G^{\prime }\left({\text{d}}_{i}\right)\in {\mathbb{R}}^{P\times Q}$.

### 3.3 Threat Model

The Federated KDE setting involves one server and several users. From a *utility* point of view, the functional role of the server is to define the parameters provided to the users, receive the users’ functions evaluations on all points of the grid, and estimate the density surface via aggregation. The functional goal of the users is to assist in the computation of the density surface while keeping their location data local. The server must specify the following parameters in Federated KDE: (i) the P×Q grid of query points and (ii) the kernel bandwidth h. Each user evaluates the kernel function at every coordinate on the grid with the bandwidth specified by the server, and sends back to the server the function evaluation. In addition, if Federated RFF KDE is used, the server must also specify (iii) the number of random features B to be used by users. Each user picks their own B features randomly (the server only controls the number not the selection of random features), and uses those same features consistently to evaluate the function on the grid points, whenever it is asked by the server.

From a *privacy* point of view, we consider a *malicious server*: in addition to computing the density surface, it also wants to infer the users’ locations from the received function evaluations on the grid, whether these are exact in Federated KDE or projections in Federated RFF KDE. We do not assume any limits on the server’s computational resources. Users’ location data can still be inferred by a malicious server in Federated KDE even though users’ data are not directly disclosed, as discussed in Fig. 2. We show that this location inference is prevented when Federated RFF KDE is used: the users assist the computation by truthfully responding to the server’s queries, but they use random projections to prevent inference of their location. We assume that the server can make one round of queries or multiple queries with different choices of B and h. However, we also assume that users can refuse to answer queries for values of B and h that fall outside a pre-specified policy range (defined below) that is known *ex ante* to all parties (as in Lemma 3). The need for such a policy can be appreciated by seeing how the Federated KDE without such constraints can disclose users’ locations to the server, along the lines discussed in Fig. 2. Such policy is proposed as an improvement to the way the protocol handles updates (see Alg. 3). The privacy analysis is provided in section 3.5: it shows that the malicious server cannot accurately infer the users’ location even if allowed to make an arbitrary number of queries at any number of spatial locations.

### 3.4 Convergence Analysis

In this section, we demonstrate the statistical justification behind Federated RFF KDE, and show that it provides consistent inference under weak regularity conditions that are automatically satisfied in real settings. Considering any coordinate ${\text{g}}_{pq}$ on the P×Q grid, the complete-data density estimator on it is

(8) $$f\left({\text{g}}_{pq}|\text{D}\right)=\frac{1}{N}\overset{N}{\underset{i=1}{\sum }}k_{h}\left({\text{g}}_{pq},{\text{d}}_{i}\right).$$

Assuming the kernel k has properties stated in section 2.3, (8) can be written as

(9) $$f\left({\text{g}}_{pq}|\text{D}\right)\overset{\left(2\right)}{=}\frac{1}{N}\overset{N}{\underset{i=1}{\sum }}E\left[e^{j\omega^{⊤}\left({\text{g}}_{pq}-{\text{d}}_{i}\right)}\right]\;\overset{\left(3\right)}{=}\frac{1}{N}\overset{N}{\underset{i=1}{\sum }}E\left[\phi^{i}\left({\text{g}}_{pq},{\text{d}}_{i}\right)\right]$$

Define $\mu_{i}$ as an unbiased estimator of user i’s kernel evaluation. We have already seen an example of such an estimator: the sum of i’s data projection onto B randomly chosen basis functions. Using this estimator then gives us

(10) $$E\mu_{i}\left({\text{g}}_{pq},{\text{d}}_{i}\right)=E\left[\phi^{i}\left({\text{g}}_{pq},{\text{d}}_{i}\right)\right]\approx \frac{1}{B}\overset{B}{\underset{b=1}{\sum }}\phi_{b}^{i}\left({\text{g}}_{mn},{\text{d}}_{i}\right).$$

Note further that convergence of μ to the target expectation as B→∞ is guaranteed under the law of large numbers (the conditions of which are satisfied for the Fourier basis of the Gaussian kernel).

Combining (9) and (10), we obtain the local estimator

(11) $$f^{\prime }\left({\text{g}}_{pq}|\text{D}\right)=\frac{1}{N}\overset{N}{\underset{i=1}{\sum }}\mu_{i}\left({\text{g}}_{pq},{\text{d}}_{i}\right)$$

Next, define I as a uniform random variable from (1,2, … ,N), and η as an unbiased estimator of $\mu_{I}$ such that

(12) $$E\eta \left({\text{g}}_{pq}\right)=E\left[\mu_{I}\left({\text{g}}_{pq},{\text{d}}_{I}\right)\right]=\frac{1}{N}\overset{N}{\underset{i=1}{\sum }}\mu_{i}\left({\text{g}}_{pq},{\text{d}}_{i}\right)$$

Then by substituting (12) into (11), we get

(13) $$f\left({\text{g}}_{pq}|\text{D}\right)\approx f^{\prime }\left({\text{g}}_{pq}|\text{D}\right)=E\eta \left({\text{g}}_{pq}\right)$$

Examining (12) and (13), one notes that η has the same expectation as $\mu_{i}$, and by the law of large numbers, as N→∞, $f^{\prime }\left({\text{g}}_{pq}|\text{D}\right)$ converges to $f\left({\text{g}}_{pq}|\text{D}\right)$. More importantly, this property always holds so long as μ is an unbiased and consistent estimator of $\phi^{i}$. In particular, *whatever* the choice of B in (10) is (including B=1), convergence in N will hold. Thus, the proposed method allows us to obtain a consistent approximation to the complete-data solution, while using only minimal and spatially delocalized information from each user.

Another insight from (13) is that there is a precision tradeoff between users and basis functions. For a fixed location d, adding an additional user or an additional basis function will have a similar effect. On the one hand, this embodies the price that is paid for maintaining privacy: each user contributes less information to the final solution, and more users are hence required when B is small. Since, however, convergence of the sample mean exhibits diminishing returns to sample size (e.g., the $1/\sqrt{n}$ scaling of the standard error of the mean), we may also expect that the first few basis functions from any given user will contribute the largest gain in precision, and we may hence get much of the informational benefit from user participation without using a large number of functions. In the next section, we consider specifically how the number of basis functions B affects privacy. In section 4, we further show that the proposed framework is able to obtain good results under realistic conditions, while still effectively concealing user locations.

#### Illustration of Convergence to KDE:

**Figure 3:**
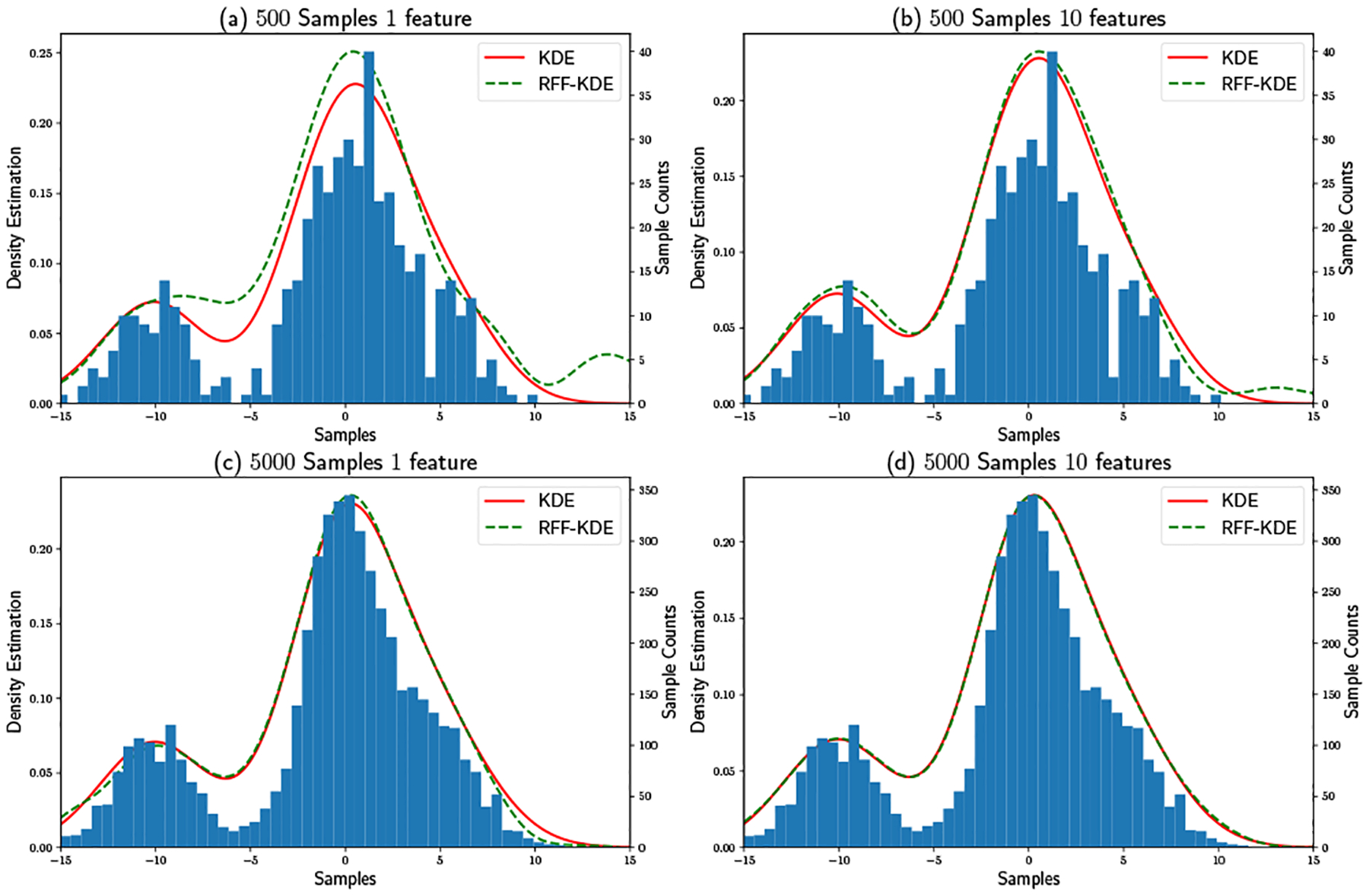
RF-KDE Converges to KDE.

Beyond the theoretical analysis of the proposed method, the following 1 dimensional example helps to further illustrate its effectiveness. In this example, observations are sampled from three independent Gaussians $𝒩\left(-10,2^{2}\right)$, $𝒩\left(0,2^{2}\right)$ and $𝒩\left(5,2^{2}\right)$ with ratio 1 : 3 : 1. Density is evaluated at 1000 points which are evenly spaced over the interval [−15, 15]. Fig 3 shows how the number of samples and number of random features affect the proposed method converging to KDE. By looking at Fig 3 (a) and (b), with a small number of samples 500, the proposed method with 1 feature cannot precisely estimate KDE results, even though the overall estimation is reasonable. When with 10 features, the estimation is close to KDE. Comparing Fig 3 (b) with (c), it is empirically shown that, an additional user and an additional basis function will have a similar effect. When both the number of samples and the number of features are high as in Fig 3 (d), the proposed method closely approximates the KDE solution.

### 3.5 Privacy Analysis

In our setting, users’ locations are never transmitted to the server, and the malicious server can only infer users’ locations based on their feature projections. Here, we show that the server cannot infer users’ locations, even given the ability to make arbitrary numbers of queries at any number of spatial locations.

#### Localization:

**Figure 4:**
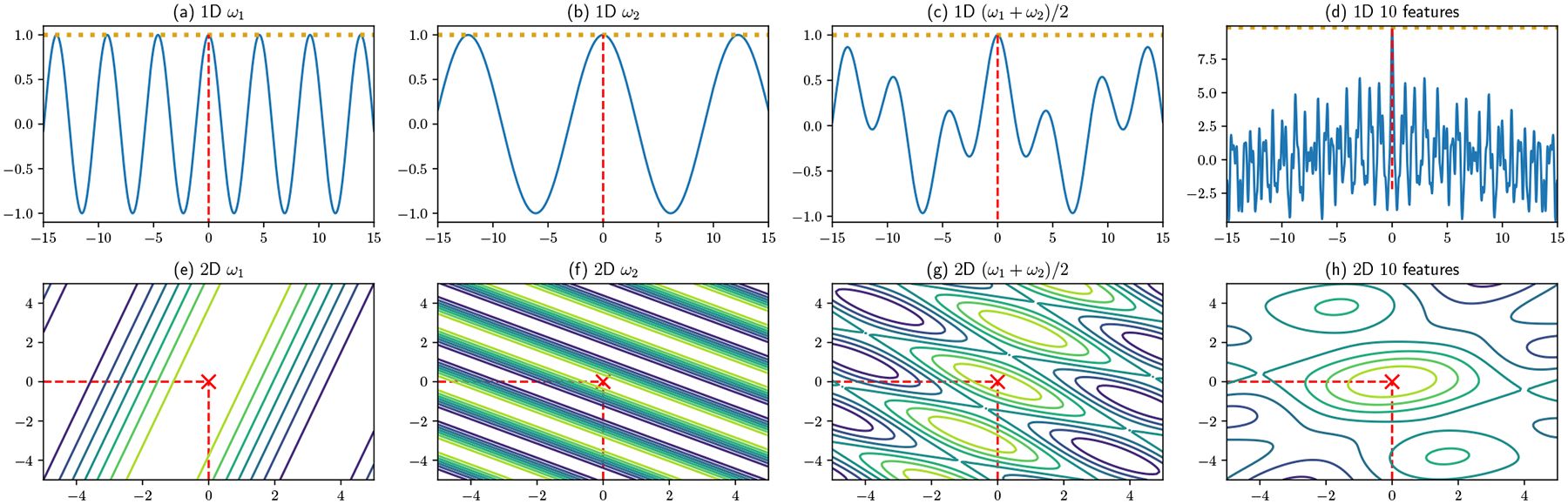
User’s response surfaces for different random vectors and numbers of random features.

Fig 4 shows the random feature projections whose values are potentially returned as responses in a one-dimensional (top) vs. two-dimensional (bottom) case. Without loss of generality, we define the user’s true location to be at 0 and (0, 0), respectively. Examples of individual projections are shown respectively in (a) and (b) and (e) and (f). Note that each maps the user’s true location to an equivalence class of possible positions, reflected in respectively the peaks of the 1D oscillatory functions and the bands of the 2D functions: given these choices of ω, any other true location on another local maximum would lead to the same function evaluation. While repeated queries by the server could build up an image of the function being used, they cannot reveal which coordinate in the equivalence class (defined in lemma 2) corresponds to the true location. When multiple features per user are employed ((c), (d), (g), and (h)), the result is still a repeating pattern, but the set of equivalent coordinates becomes more dispersed. For an area of fixed size, a sufficiently large number of features will lead to a function with only one maximum in the region, and the user will be localized. Unlike prior work, our approach thus focuses on using few functions per user, exploiting the insight illustrated in Fig. 3 that more data points can make up for using fewer features per point.

In this section, we provide a more formal characterization regarding the above intuition for how our privacy preservation scheme works, the impact of using multiple features per user, and the relationship of privacy preservation to bandwidth. In section 4, we will empirically show how the number of basis functions affects estimation performance and privacy loss under real-world conditions.

Lemma 1. *A malicious server seeking to estimate a user’s location will predict that the user resides in a location yielding a maximum on the surface formed by his/her feature projections*.

Proof. In the one feature case, user i’s basis function has the form

(14) $$\phi^{i}\left({\text{g}}_{pq},{\text{d}}_{i}\right)=\left[\text{cos}\left(\omega^{⊤}{\text{g}}_{pq}\right),\text{sin}\left(\omega^{⊤}{\text{g}}_{pq}\right)\right]{\left[\text{cos}\left(\omega^{⊤}{\text{d}}_{i}\right),\text{sin}\left(\omega^{⊤}{\text{d}}_{i}\right)\right]}^{⊤}$$

We observe immediately that $\phi^{i}\left({\text{d}}_{i},{\text{d}}_{i}\right)=1$; since $\phi^{i}\left({\text{g}}_{pq},{\text{d}}_{i}\right)\leq 1$ for all $g_{pq}$, it follows that a candidate ${\text{g}}_{pq}$ can be equal to ${\text{d}}_{i}$ only if it is a maximum of $\phi^{i}\left({\text{g}}_{pq},{\text{d}}_{i}\right)$. Now, consider the general case in which we have B basis functions. Each has the form of (14), but with different ω, and the user evaluation at point ${\text{g}}_{pq}$ yields $\frac{1}{B}\sum_{b=1}^{B}\phi_{b}^{i}\left({\text{g}}_{pq},{\text{d}}_{i}\right)$. As before, $\phi_{b}^{i}\left({\text{d}}_{i},{\text{d}}_{i}\right)=1$, and $\phi_{b}^{i}\left({\text{g}}_{pq},{\text{d}}_{i}\right)\leq 1$ for all b, ${\text{g}}_{pq}$; thus it again follows that $g_{pq}$ can be equal to ${\text{d}}_{i}$ only if it is a maximum of the surface formed by the feature projections. Any optimal prediction for ${\text{d}}_{i}$ will thus be on a maximum of the projected feature surface, irrespective of B or $\omega_{b}$. □

Is the optimal solution unique? Setting the derivative in the single-basis case $\nabla_{{\text{g}}_{pq}}\phi^{i}\left({\text{g}}_{pq},{\text{d}}_{i}\right)$ to be 0, we obtain

(15) $$\text{tan}\left(\omega^{⊤}{\text{g}}_{pq}\right)=\text{tan}\left(\omega^{⊤}{\text{d}}_{i}\right),$$

the solutions to which are candidate location predictions. However, the solutions to (15) are non-unique, as any g that satisfies $\omega^{⊤}\text{g}=\omega^{⊤}{\text{d}}_{i}+2t\pi$ also satisfies (15), where t can be any integer. This generalizes to the multiple feature case: setting $\nabla_{{\text{g}}_{pq}}\sum_{b=1}^{B}\phi_{b}^{i}\left({\text{g}}_{pq},{\text{d}}_{i}\right)$ to 0, then for each $\omega_{b}$, (15) holds. So the maxima are obtained when $\omega_{b}^{⊤}\text{g}=\omega_{b}^{⊤}{\text{d}}_{i}+2t\pi$ with more than two $\omega_{b}$’s, b∈{B}. These are the gs that appear as “peaks” in Fig 4 (g) and (h). In (g), since there are only two features, all peaks will have the same function evaluation; However, in (h), different peaks consists of different number of satisfied ω’s, with one equivalence class (including the true location) having the maximum value, and others having lower values. These intersecting constraints gradually reduce the solution set, allowing the user to be increasingly well-localized.

As this suggests, the equivalence class of coordinates having optimal prediction solutions can be characterized. For a single basis function, we state this as follows:

Lemma 2. *Let* x *be any location to be evaluated, and let D(x) be the equivalence class of locations that cannot be distinguished by distinct query responses for a user using a single basis function with frequency ω. Then*

$$\text{D}\left(\text{x}\right)=\left\{\text{y}|\text{y}=\text{x}+\alpha \frac{2\pi }{∥\omega {∥}^{2}}\omega +\text{u},\forall \alpha \in \mathbb{Z}\right\}\;\text{ where }\omega {\text{u}}^{⊤}=0$$

Proof. Let u be any vector which satisfies ${\text{u}}^{⊤}\omega =0$, and then $\omega^{⊤}\left({\text{g}}_{pq}+\text{u}\right)=\omega^{⊤}{\text{g}}_{pq}$. Therefore, with (14), $\phi^{i}\left(\text{x},{\text{g}}_{pq}+\text{u}\right)=\phi^{i}\left(\text{x},{\text{g}}_{pq}\right)$ always holds. Next, let α be any integer. Obviously, $\omega^{⊤}\left({\text{g}}_{pq}+\alpha \frac{2\pi }{∥\omega {∥}^{2}}\omega \right)=\omega^{⊤}{\text{g}}_{pq}+2\pi \alpha$. Therefore, by the periodic property of trigonometric basis function, $\phi^{i}\left(\text{x},{\text{g}}_{pq}+\alpha \frac{2\pi }{∥\omega {∥}^{2}}\omega \right))=\phi^{i}\left(\text{x},{\text{g}}_{pq}\right)$ holds. □

In the case of multiple features associated with frequencies $\omega_{1},\;\ldots \;,\omega_{B}$, the corresponding equivalence class is trivially $\cap_{i=1}^{B}D_{i}(\text{x})$, where ${\text{D}}_{i}\left(\text{x}\right)$ is the equivalence class associated with frequency $\omega_{i}$. D(x) has the cardinality of the continuum, but (setting aside cases of measure zero), its intersections are of countable size (as illustrated e.g., in Fig. 4 (g)).

Lemma 1 and 2 show that (1) optimal predictions under unlimited querying are limited to maxima of the projected feature surface, and that (2) these maxima are in general spatially delocalized. Thus, the server cannot in general recover users’ locations, even given unlimited queries. That said, increasing the number of basis functions per user reduces the size of the equivalence class, resulting in ever more widely spaced maxima. When users have already been localized to an initial polygon (i.e., the requested area), this will eventually localize them. It is thus important to keep the number of features small. The bandwidth is also relevant in this finite-area case, as we now discuss.

#### Maximum Bandwidth:

**Figure 5:**
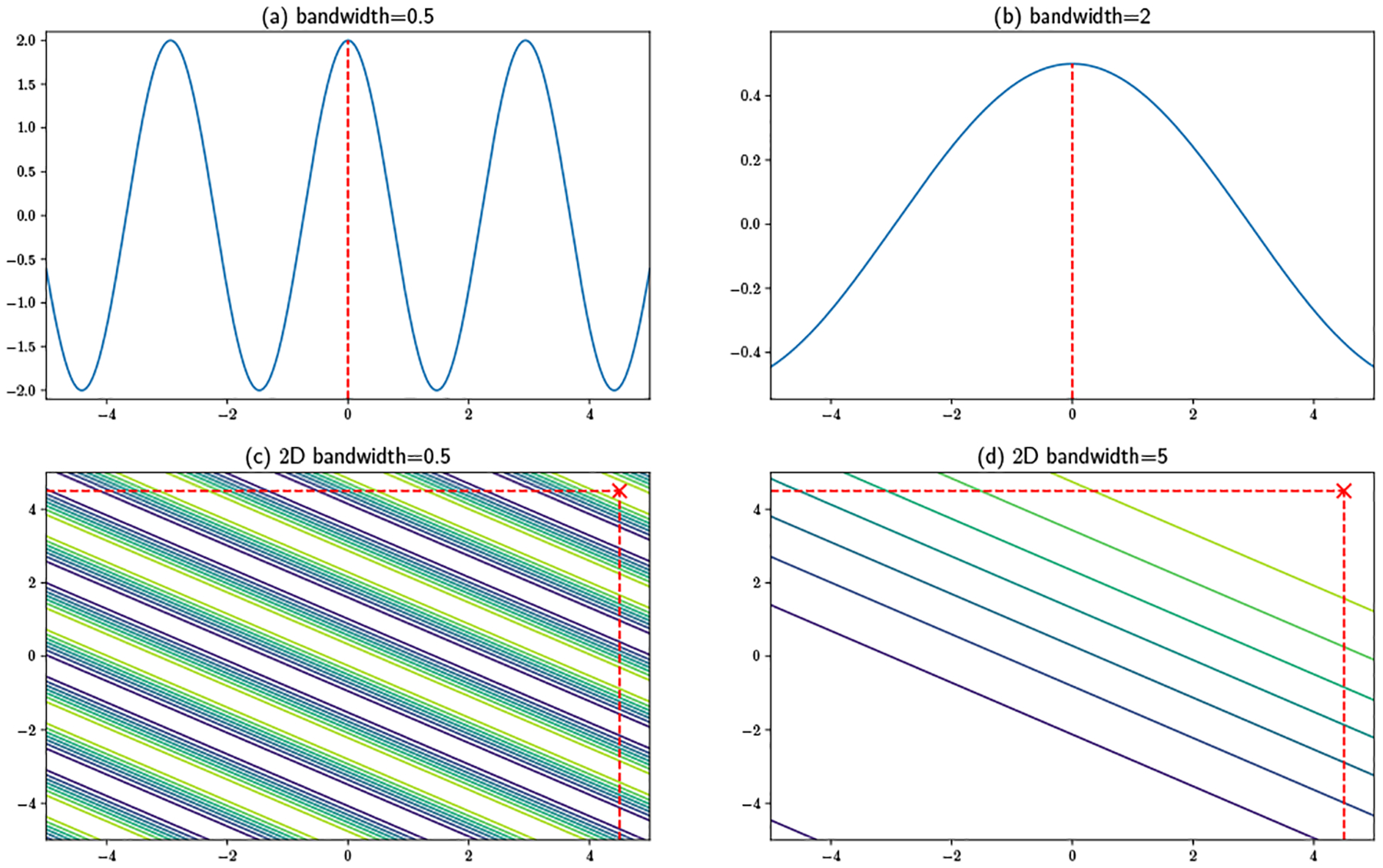
Maximum bandwidth for spatial delocalization.

Even though the server in the single-feature case cannot localize a user beyond a set of bands containing possible locations, one can see in Fig. 5 that bandwidth selection will influence the number of bands appearing in the area. Intuitively, with a larger bandwidth, the number of bands is smaller. A larger number of bands translates to a larger range of possible user locations, and hence better privacy preservation. We illustrate this in Fig. 5, with panels (a) and (b) showing features with bandwidths 0.5 and 2 for a user located at the origin. With a smaller bandwidth, multiple equivalent local maxima appear within the focal region. However, only a single local maximum can be found with the larger bandwidth, allowing the server to potentially infer the user’s location. Panels (c) and (d) show a 2D example, here with a user located at (4.5, 4.5), which is at top right corner of the region. With the smaller bandwidth (0.5), multiple bands run across the region, which makes it infeasible to determine the user’s location. A much larger bandwidth (5) leads to a single band peaking in the top right corner, making it clear that the user must reside in this region. This phenomenon implies that a bandwidth that is relatively small compared to the size of the estimation region is to be preferred in a privacy preservation scenario. Fortunately, such bandwidths are usually optimal from an estimation standpoint, and optimal bandwidths decline with sample size. A maximum size does not therefore impair convergence in the large data limit. A formal criterion for determining the maximum acceptable bandwidth can be constructed based on the risk of having a small number of bands appear, which can be bounded in the two-dimensional case by exploiting the band structure and isotropy of the random features. Specifically:

Lemma 3. *In the 2D, one feature case, assume the largest inscribed square in A has side length* l, *and* C(x) *is the CDF of the chi-squared distribution. To ensure at least* j *bands to appear in the area with no less than* 1−C(γ) *probability, bandwidth* h *should be selected smaller than* $\frac{\sqrt{\gamma }l}{2\pi j}$.

The proof of this lemma is provided in Appendix A.1. The derived bandwidth is simple and intuitive: it is linearly bounded by both the region’s side length and the inverse of the number of bands expected to appear, and γ is a tunable parameter to control how tight the bound is. With a large side length or smaller number of bands, a relatively large bandwidth can be used. On the other hand, when the estimation region is small or more bands are expected, a smaller bandwidth is preferred. Note that this bound is for the worst data distribution with the worst projection vector samples. Specifically, this protects users in corner and edge areas, such as Fig 5 (c) and (d). If only one or two bands appear when the user is at these regions, the server will potentially be able to localize the user at a small area. Obviously when there are at least 3 bands, users’ locations are well protected, since there will be at least one band across the central area with length at least l, so j should not be smaller than 2. In fact, this is an extreme example and real applications are generally more favorable: numerical tests in section 4 show that any statistically reasonable bandwidth for real application will be enough for privacy preservation purposes. However, having the guideline of Lemma 3 gives the user the ability to recognize and refuse to compute solutions for bandwidths that could lead to unacceptable risk, without that refusal revealing anything about the user’s location (since the resulting bandwidth constraint depends only on the target area and risk tolerance).

The proposed framework also needs to avoid privacy leakage from any user’s projections with or without distinct bandwidths, since the server can query users for updates multiple times whether the user is moving or being still, or may employ different bandwidths, h. As above, we focus on policies that can be unilaterally enforced by users.

#### Multiple Queries:

**Figure 6:**
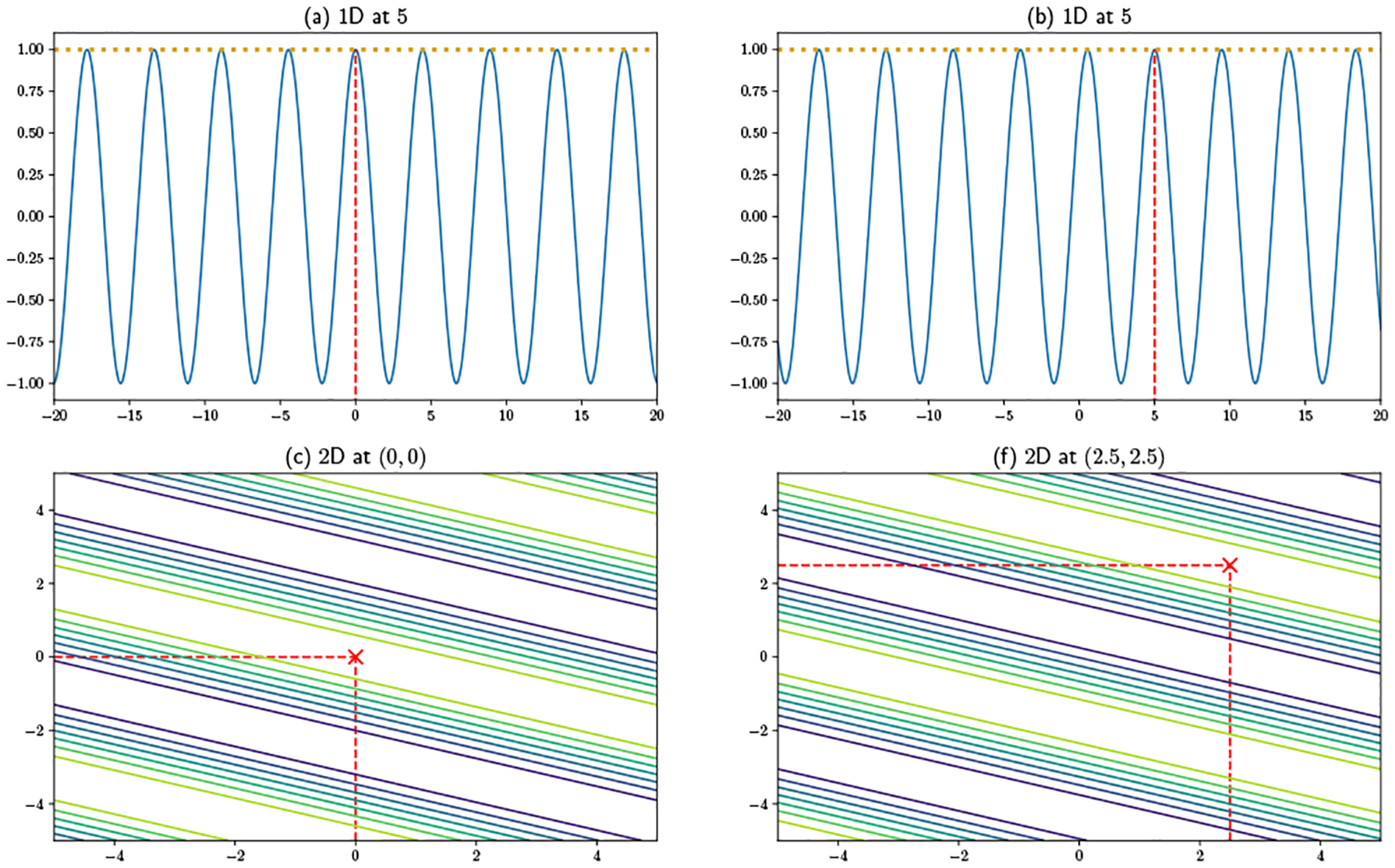
Projection surfaces for queries to a moving user.

For multiple queries to the user at the same location ${\text{d}}_{i}$, so long as the user employs the same random vector ω, she/he always generates the same projection surface with (7). Thus, no matter how many queries are made, the server cannot learn more than the user’s random feature projections. Fig 6 shows examples of moving users responding with the same projection vectors. In the 1D case (a) and (b), a user moves from 0 to 5. One can observe that in (b), as the user moves, all local maxima are also moving simultaneously. Thus, the server cannot identify the start and end points of the user’s travel. In addition, the server cannot even figure out the direction of the user’s travel. Therefore, it is infeasible for the server to figure out the relative offset of the user’s travel. In the 2D case (c) and (d), a user moves from (0, 0) to (2.5, 2.5). Similarly, the overall pattern is moving simultaneously as the user moves. All observations from 1D case still hold here. Therefore, for both static and moving cases, as long as a user always uses the same random vector, this user’s travel track and relative offset cannot be inferred by the server.

#### Bandwidth Rescaling:

The key to dealing with the second issue (multiple bandwidth queries) is that each user generates ω only once and stores it locally and the direction of ω is fixed. After rescaling ω with $\frac{1}{h}\omega$, only the period is changed, but not the direction. Therefore, rescaling ω has the effect of “shrinking or amplifying” the projection surface. With this trick, the server still cannot reveal a user’s location by querying them for evaluations with different bandwidths, and the worst case is that the server can locate the band on which the user resides. This is the worst case because the band on which the user resides will not shift, and all the other bands may shift when using different bandwidths. If the server makes queries with different bandwidths, the server can potentially infer that the band that does not shift when changing bandwidth is the one with the user’s location. To solve this problem, we require that several invariant bands remain present on the projection surfaces associated with different query bandwidths, ensuring that the server cannot reliably localize the user to a single band. We accomplish this by employing a bandwidth rescaling policy that requires bandwidths to be selected from a set of specific discrete values as shown in lemma 4 and Alg. 3. So long as $\omega_{b}^{i}$ satisfies lemma 3 and is derived from Alg. 3, it can be ensured that multiple invariant bands remain across the area. This provides privacy preservation against an attack of localizing a user on a single band, even though one band can still provide considerable privacy protection. As with the maximum bandwidth constraint, this is an *ex ante* policy that can be enforced by the user.

Lemma 4*. In the one feature case, assume that a user samples ω from $\frac{1}{h_{0}^{2}}𝒩\left(0,\text{I}\right)$, where $h_{0}$ is a properly small bandwidth, and the number of bands with bandwidth $h_{0}$ is m. By rescaling bandwidth $h=\left(4n+1\right)h_{0}$ where n≥1 is an integer, there will be at least $\left\lfloor \frac{m}{4n+1}\right\rfloor$ bands, whose locations are overlapping with a subset of the m bands*.

The proof of this lemma is provided in Appendix A.2.


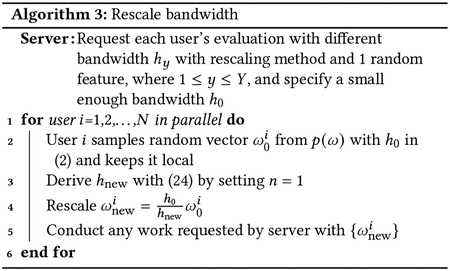


**Figure 7:**
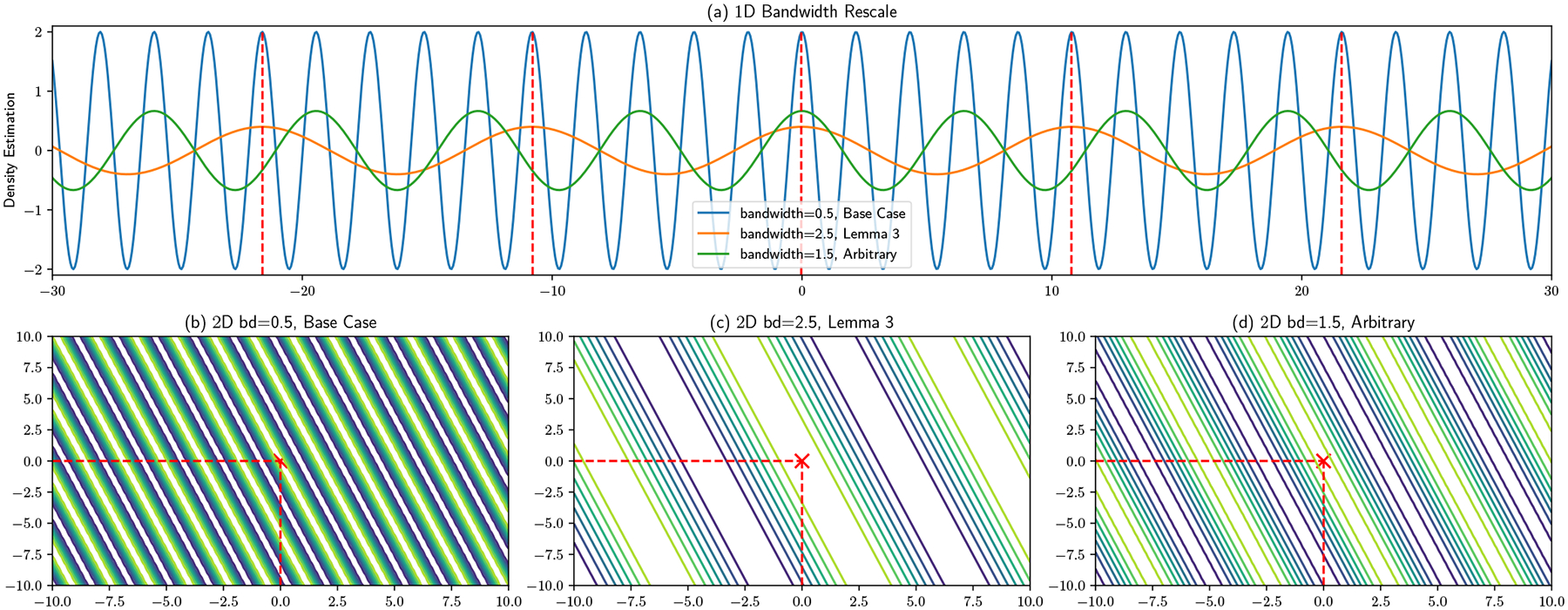
Bandwidth rescaling.

Fig 7 shows both 1D and 2D examples of bandwidth rescaling with and without Lemma 4. In Fig 7 (a), if we set the base bandwidth as 0.5, then the next bandwidth selected by lemma 4 is 2.5. One can observe that projection surfaces estimated with these two bandwidths have duplicate local maxima, and they appear periodically. Therefore, if the server queries the user for projection surfaces with two different bandwidths chosen via lemma 4, the two response surfaces will always have several invariant bands, which makes it impossible to further localize the user. However, if an arbitrary bandwidth such as 1.25 is used, the server can easily eliminate the possibility of data being at some of local maxima, since they are not at the same location as the base case. Even though there are still multiple bands for each bandwidth, those that shift are not effective for protecting the user’s privacy against an attentive server. Note that another arbitrarily selected bandwidth might lead to an even worse case. In the 2D case of Fig 7 (b) and (c), by setting n=1 in lemma 4, an overlapping band appears every 5 bands. But in (d), some of bands do not overlap with any band in (b). The server will not localize the user to these locations, which means these bands do not provide any protection for the user’s location.

### 3.6 Complexity

As a non-parametric method, Federated RFF KDE is light enough to be implemented on edge devices. To analyze its complexity, (7) shows B basis functions evaluated at query points ${\text{g}}_{pq}$. So there are a total of PQB basis functions for every user. Each basis function is a problem with fixed size as shown in (4). So the overall complexity on user side is 𝒪(PQB). Obviously, the complexity of a single user conducting Kernel Density Estimation is 𝒪(PQ) on the same grid. Therefore, when using few random feature or even only 1 feature, the complexity of the proposed framework is almost the same as KDE. This beats other location protecting mechanisms which are based on adding noise, which incurs additional calculation costs.

### 3.7 Comparison with Spatially Local Noise

**Figure 8:**
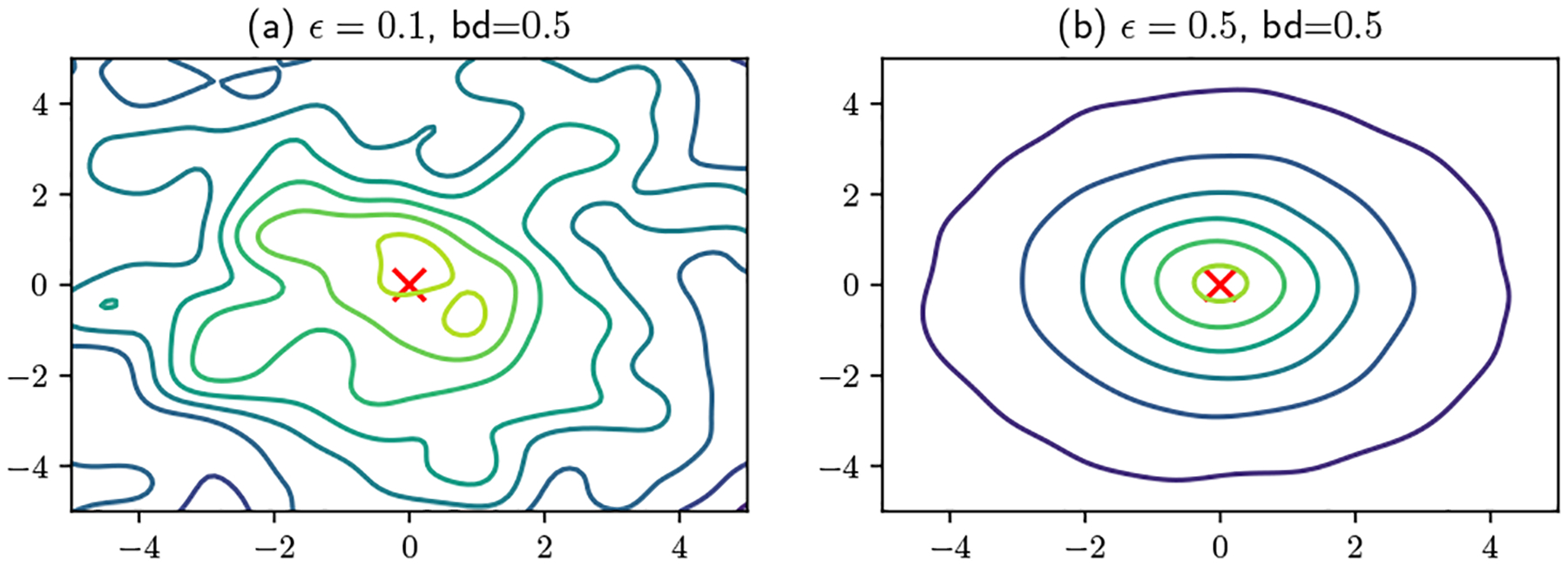
Visualization of GeoInd-DP [1].

In section 1, a 1D discrete example Fig 1 was used to show the distinctions between the ideas behind local noise-based methods and the proposed method. Fig 8 shows a 2D example of a local noise (DP) method for location-service, Geo-indistinguishability (GeoInd-DP) [1], which is the approach to which we compare Federated RFF KDE in our empirical experiments. Here we assume a data point at (0, 0), running GeoInd-DP 50, 000 times independently, and then visualizing the artificial noise distribution with KDE. Parameter ϵ is set to 0.1 and 0.5 for (a) and (b), respectively. From the figure, one can observe that even though user’s precise location is not shared, most of processed data points are located around the ground truth. Therefore, the server can localize the user’s location to a smaller region instead of all over the map, as governed by the noise parameter ϵ.

## 4 EXPERIMENTS

### 4.1 Datasets

Federated RFF KDE method is evaluated on both synthetic and real-world datasets.

#### 4.1.1 Synthetic Data.

We first demonstrate our method on two synthetic population distributions, one constructed for uniformity and the other for heterogeneity:

1. A mixture of 9 independent Gaussians with means from all possible pairs ${\left(i,j\right)}^{⊤}$ where i∈{−1,0,1} and j∈{−1,0,1} and each with diagonal covariance $\Sigma_{ii}=0.25$.
2. A mixture of 8 Gaussians arranged in an octagon with component mean $\mu_{i}=\left(3\text{ cos}\left(\pi i/4\right),3\text{ sin}\left(\pi i/4\right)\right)$, and covariance
$$\Sigma_{i}=\left[\begin{array}{cc} {\text{cos}}^{2}\frac{2i}{4}+0.16^{2}\;{\text{sin}}^{2}\frac{\pi i}{4} & \left(1-0.16^{2}\right)\text{sin}\frac{\pi i}{4}\text{cos}\frac{\pi i}{4}\\ \left(1-0.16^{2}\right)\text{sin}\frac{\pi i}{4}\text{cos}\frac{\pi i}{4} & {\text{sin}}^{2}\frac{\pi i}{4}+0.16^{2}{\text{cos}}^{2}\frac{\pi i}{4} \end{array}\right]\text{ for }i\in \left\{1,\;\ldots \;,8\right\}.$$

**Figure 9:**
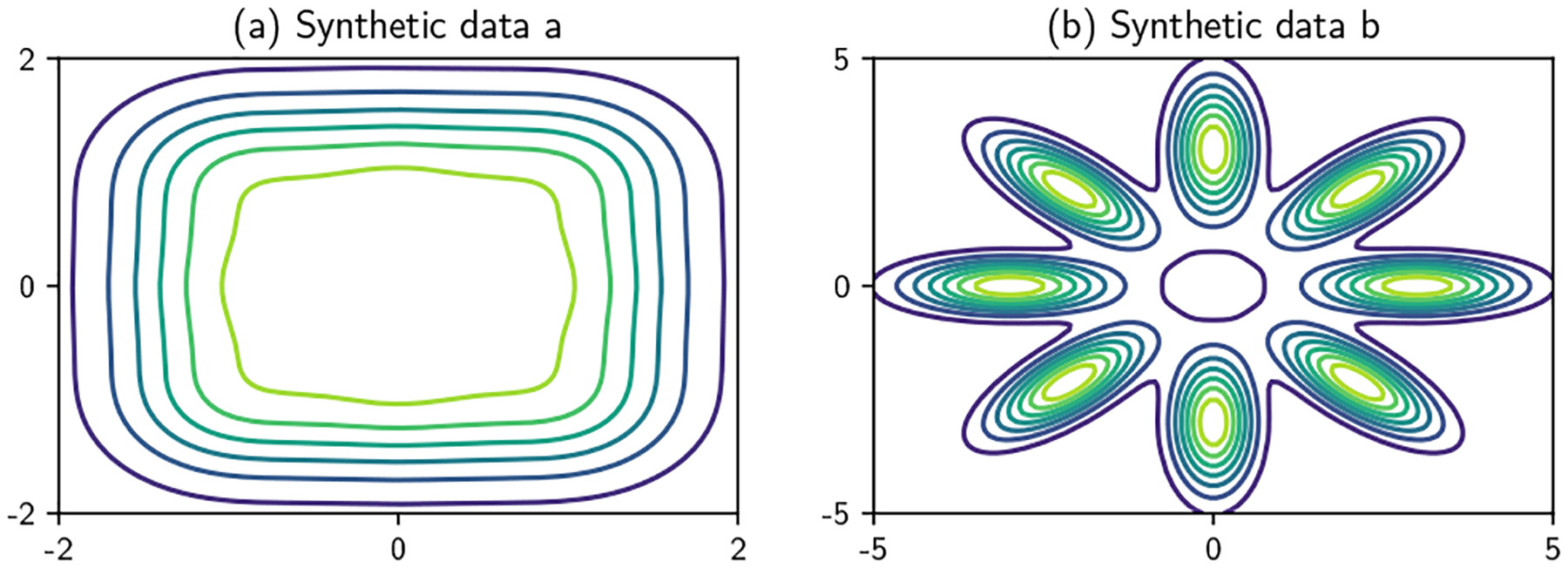
True function surfaces for the synthetic data.

The densities of these synthetic datasets are shown in Fig 9.

#### 4.1.2 Real-World Data.

To evaluate our model on real-world data, we use two location-based social networks, Gowalla and Brightkite^2^ [11, 30]. Gowalla contains a total of 6,442,890 user check-ins over the period of Feb. 2009 - Oct. 2010, and Brightkite contains 4,491,143 checkins over the period of Apr. 2008 - Oct. 2010. Each check-in record consists of the location represented by a tuple of latitude, longitude, user ID and check-in time. For population density estimation purposes, we use only the location (latitude, longitude) of the check-ins. To evaluate the proposed method in realistic urban settings, we employ the downtown areas of several major cities. For Gowalla, we selected LA, London and Chicago, and for Brightkite, we selected LA, Tokyo and Chicago. All check-ins within these selected areas are used to estimate the target density, with each check-in treated as belonging to an independent virtual user for purposes of our analysis. The detailed information about area selection and density estimation is listed in Appendix B.1.

### 4.2 Model Evaluation

#### 4.2.1 Performance.

To measure estimation performance, we use the Spearman (rank) correlation [26] to compare our estimated density surfaces with the ground truth. After evaluating two density surfaces at each coordinate ${\text{g}}_{pq}$ on the grid with the same set of observations D as $\left\{f^{\prime }\left({\text{g}}_{pq}|\text{D}\right)\right\}$ and $\left\{f\left({\text{g}}_{pq}|\text{D}\right)\right\}$ where 1≤p≤P and 1≤q≤Q, we calculate rank correlation between them to measure difference in the distributions. For the synthetic datasets, since the ground truth function is known, it is feasible to compare estimation directly with the ground truth. For real-world datasets, we take the complete-data KDE to be the ground truth for evaluation purposes, as this reflects the estimate that could be obtained by pooling all available data, with no privacy limitations. In other words, since the proposed method is expected to converge to KDE in utility and protect user’s privacy at the same time, beating KDE in utility is not our objective. As described below, we examine the rank correlation of our method (and of GeoInd) with the ground truth over a range of privacy settings (for our method, choices of B, for GeoInd, choices of ϵ); an ideal method would produce a correlation close to 1, indicating a nearly identical match between the shape of the inferred density and the target.

#### 4.2.2 Privacy.

Our choice of privacy metric is motivated by Fig. 4. Because the density surface available to the server is globally delocalized, the server can only infer that the user lies on or near the local maximum density points or ridges among the surface. Let user i have $K_{i}$ local grid maxima, having coordinate vectors ${\text{g}}_{j}^{i}$ with $j\in 1,\;\ldots \;,K_{i}$ with local density evaluations $f^{\prime }\left({\text{g}}_{j}^{i}|{\text{d}}_{i}\right)$ is $e_{j}^{i}$. We then define the privacy score $Z_{i}$ of user i by

(16) $$Z_{i}=\frac{1}{K_{i}}\sum_{j=1}^{K_{i}}e_{j}^{i^{\prime }}{\left\|{\text{g}}_{j}^{i}-{\text{d}}_{i}\right\|}_{2}$$

where $e_{j}^{i\text{'}}=\frac{e_{j}^{i}}{\sum_{j=1}^{K_{i}}e_{j}^{i}}$ is a normalized weight reflecting the strength of evidence for i residing near location ${\text{g}}_{j}^{i}$. $Z_{i}$ is thus the expected error (in units of distance) for the server attempting to guess ${\text{d}}_{i}$ on the basis of i’s basis projection. We likewise score the privacy level of the whole system by the average privacy score: $Z=\frac{1}{N}\sum_{i=1}^{N}Z_{i}$. We employ normalization weights $\left(e_{j}^{i}\right)$ in (16) to account for differences in the height of maxima and for true maximum/grid non-alignment (which can make the global maximum, if unique, an imperfect predictor). However, to prevent the server from placing weight on inferior local maxima, we filter local maxima via another parameter ϵ to remove those with lower levels of $f^{\prime }$ : assuming the global maximum on user i’s surface is $e_{\text{max}}^{i}$, one local maximum will be considered if and only if $e_{j}^{i}\geq \frac{e_{\text{max}}^{i}}{\epsilon }$. In all following numerical tests, ϵ is set as 1.1. Simply put, the privacy metric in (16) is the expected error in the attacker’s prediction of the user’s location, expressed in terms of distance. Since the server can localize the user at multiple locations with different confidence in each, we take the weighted average of those distances, where the weights reflect the attacker’s uncertainty in each inferred position.

#### 4.2.3 Baselines.

To evaluate our method’s estimation and privacy protection performance, we compare vs. the following benchmarks:

##### Geo-Indistinguishability.

One alternative mechanism to provide strong privacy guarantees, specifically for location-based services is GeoInd-DP [1], defined as follows:^3^

Definition 4.1 (geo-indistinguishability [1]). *A mechanism* K *satisfies* ϵ-*geo*-*indistinguishability iff for all* x, $x^{\prime }$:

(17) $$d_{p}\left(K\left(x\right),K\left(x^{\prime }\right)\right)\leq \epsilon d\left(x,x^{\prime }\right)$$

GeoInd-DP adds 2-dimensional random local noise to each user’s location so that the server cannot distinguish the user’s exact location with high confidence. In particular, for user i, noise is added to ${\text{d}}_{i}$ before calculating (5), which is expected to shift the location of the global maximum of user’s density surface. We are using the planar Laplace mechanism [1] to achieve GeoInd. Specifically, given the parameter $\epsilon \in {\mathbb{R}}^{+}$, and the actual location ${\text{x}}_{0}\in {\mathbb{R}}^{2}$, the pdf of our noise mechanism, on any other point $\text{x}\in {\mathbb{R}}^{2}$, is:

(18) $$D_{\epsilon }\left({\text{x}}_{0}\right)\left(\text{x}\right)=\frac{\epsilon^{2}}{2\pi }e^{-\epsilon d\left({\text{x}}_{0},\text{x}\right)}$$

where $\frac{\epsilon^{2}}{2\pi }$ is a normalization factor. We call this function planar *Laplacian centered* at ${\text{x}}_{0}$. The parameter settings in the experiments are as follows: for synthetic data, ϵ is from [0.6, 0.7, 0.8, 1, 3, 5, 10]. And for real-world data, ϵ takes values [10, 15, 50, 100, 500, 1000].

##### Federated KDE.

As a worst-case privacy baseline, the way to conduct Federated KDE and privacy loss measurement is as Alg. 1 and (16). Obviously, a user’s evaluation in the Federated KDE method will only have one maximum. As shown in Fig. 2, the global maximum of a user’s function evaluation sent to the server in Federated KDE is revealing, so the privacy loss of Federated KDE is a baseline: no privacy is preserved. This is the worst case for privacy preservation. As performance baseline, since Federated RFF KDE is a approximation of Federated KDE, the latter serves as a *best* case. Our goal is thus to approach the best-case estimation performance of the zero-privacy solution while still preserving privacy.

##### 0 Features.

In this best-case privacy baseline, the user responds to all queries with a constant. So the server cannot do better than guessing that the user has equal probability to be located at any grid coordinate. In this case, the privacy loss of user i is defined as the average over distance between user i’s location ${\text{d}}_{i}$ and each coordinate ${\text{g}}_{pq}$. Clearly, no method can achieve better privacy protection than this; however, the flat 0-feature “estimate” of the surface is also uninformative (and thus a worst-case estimator). Our goal is thus to approach the best-case privacy performance of the 0-feature solution, while still maintaining good estimation performance.

#### 4.2.4 Experimental details.

For bandwidth selection purposes, it is assumed that 10% data points are randomly selected by the server. Bandwidth is selected as the average kth nearest neighbor distances of all data points. Specifically, define ${\text{k}}_{i}$ as data point ${\text{x}}_{i}$’s the kth nearest neighbor, and the selected data points’ indices are M, where $\left|\text{M}\right|=\frac{N}{10}$. With the selected data, the average of kth nearest neighbor distances of all data points can be approximated by $\frac{1}{\left|\text{M}\right|}\sum_{i=1}^{\left|\text{M}\right|}\left\|{\text{k}}_{{\text{M}}_{i}}-{\text{x}}_{{\text{M}}_{i}}\right\|$. From our numerical tests, setting k as 200 for synthetic data and 500 for real-world data will achieve reasonable results. In other real-world applications, as long as following the restrictions in 3 and 4, the server can also specify whichever bandwidth that is appropriate for downstream jobs.

For the synthetic data, we analyze how the number of users N and the number of basis functions B affect estimation performance and privacy preservation. Specifically, for the two synthetic patterns in 4.1.1, we generate N∈{1000,5000,10000,20000} samples from underlying functions, with the number of basis functions is varied as B∈{1,2,3,4,5,6,7,8,50}. Since the ground truth function is known for synthetic data, rank correlation between the estimated density surface and the ground truth function surface can be used to measure the estimation performance. The privacy reveal of the training set is averaged over all users’ privacy scores per (16). For real-world data, the only difference in experimental settings is that, since the ground truth distribution is unavailable, the rank correlation is calculated between privacy preservation methods and Federated KDE on the full (pooled) dataset. To evaluate the success of a privacy preservation method, we wish the estimated density surface to match that estimated from Federated KDE as closely as possible, while also providing as little privacy loss as possible vis a vis the 0-Feature baseline.

**Figure 10:**
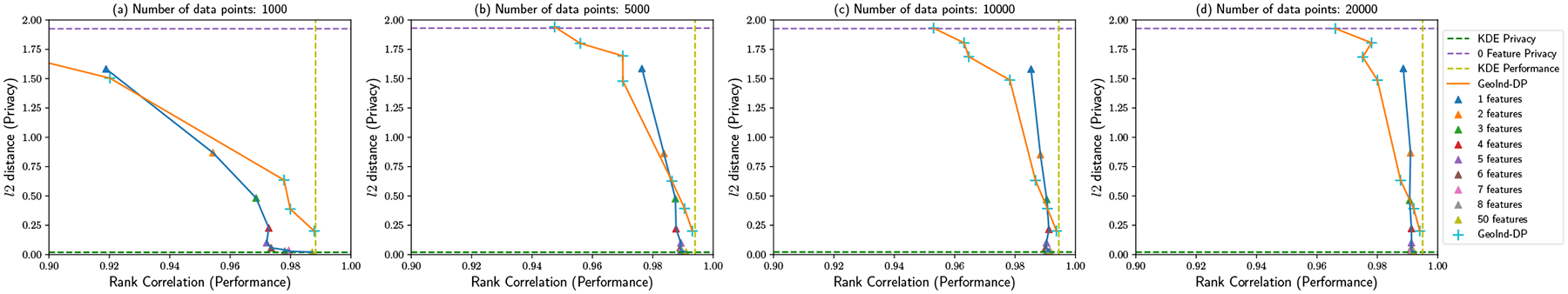
Synthetic (a) Performance and Privacy Trade-off

**Figure 11:**
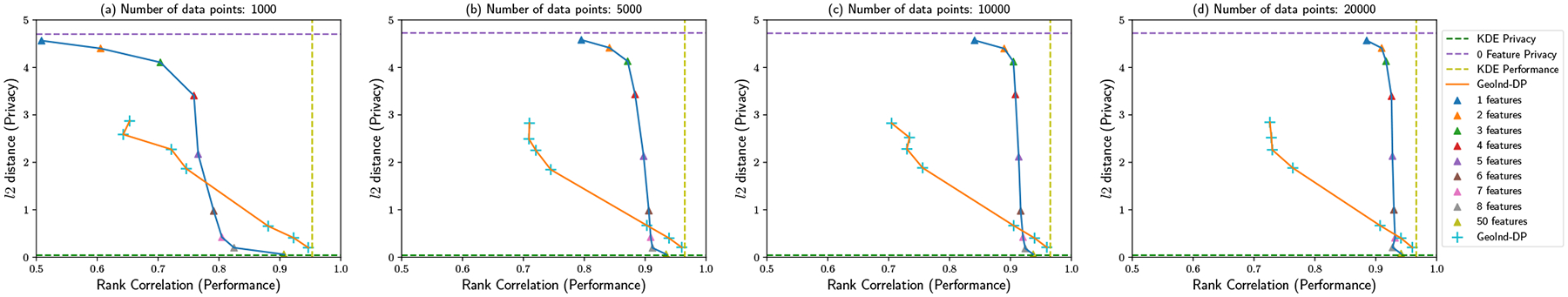
Synthetic (b) Performance and Privacy Trade-off

The results of the proposed method on synthetic cases (a) and (b) are shown in Fig. 10 and Fig. 11. For synthetic data (a), a small number of basis functions is able to achieve good estimation performance, with the rank correlation with ground truth being greater than 0.9 with only a single basis function. As for synthetic data (b), a relatively large number of either features or samples is required to achieve a reasonable result as shown in Fig. 11. This is because of the more complex surface in (b) vs. (a). In (b), with 1000 samples, the performance score is only over 0.9 a little even with 50 random features. As a comparison, when there are 20, 000 samples, only one feature can have the performance score close to 0.9. In addition, the privacy loss is only related to the number of features and the size of estimation region, but not the number of samples. This observation verifies our analysis of section 3.3: good estimation can be achieved by more users joining even with fewer features.

As for GeoInd-DP, except for the small-N (e.g., N=1000) case, it typically requires a greater privacy loss to achieve the same estimation performance as Federated RFF KDE (overtaking only when the number of features per user is very high, and neither method preserves privacy). One observation of GeoInd-DP is that, for synthetic data (a), with noise leading to the same privacy preservation as 0 features, it still gets a reasonable estimation. On the other hand, in synthetic data (b), GeoInd-DP’s utility is significantly worse than Federated RFF KDE when privacy preservation is high. The reason is synthetic data (a)’s distribution is smoother, and more uniform in high density areas than (b). In this case, the perturbation from artificial noise added to the data hurts (b) more than (a).

For both synthetic cases, there is a trade-off between privacy and estimation performance: by varying the number of basis functions, the proposed framework can balance the amount of privacy protected and estimation quality. The ideal choice can thus be tuned based on the requirements of the application. We note in passing that the bandwidth h is set as 0.55 for both datasets, allowing us to demonstrate the relationship between scale and bandwidth stated in section 3.5. With the same bandwidth, one feature privacy loss in (a) is worse than that in (b) compared with the 0 feature case. The reason is that, as stated in lemma 3, when the ratio between bandwidth and scale is larger, the number of bands across the area is usually smaller. But the width of bands is most likely the same, so the overall locations the server can localize become less.

In all 6 real-world datasets, the trade-off trend between estimation performance and privacy loss is similar to that seen in the synthetic cases. As expected, the proposed method converges to fully pooled Federated KDE when B→∞. However, the rank correlation of our estimate with the complete-data estimate is high even with a small number of basis functions, showing that fairly minimal projections can still show good performance in realistic conditions. In addition, we find that the privacy loss in the one-feature case is close to the 0-feature best case, suggesting extremely good privacy protection. Moreover, even with 3 to 5 features, privacy protection is still several hundred meters for these cases, a large displacement in the context of a dense urban core. Similar to that of synthetic data, the trade-off curve of GeoInd-DP is typically below that of the proposed method, except for the high privacy-loss case. (We note that in some cases the GeoInd-DP privacy scores go over the 0-feature line, due to the fact that high noise levels can displace the user’s location outside the search area. These noise levels, however, lead to very poor estimation performance.)

**Figure 13:**
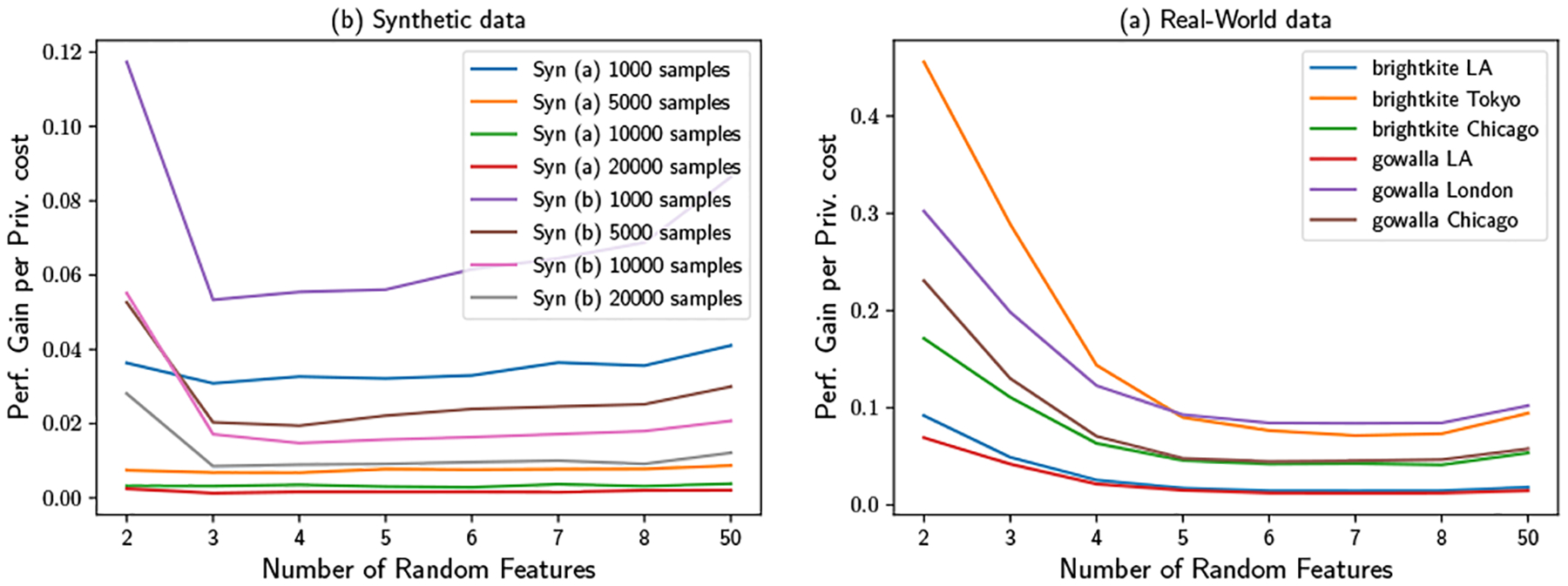
Performance gain per Privacy cost

Finally, we analyze estimation performance gain per unit privacy cost, for inclusion of multiple random basis functions after the first. The estimation performance gain for using B features is defined as its estimation performance measurement minus its performance with only one feature. Similarly, its privacy cost is defined as the privacy score with B minus that with one feature. Dividing the performance gain with privacy cost gives us the performance gain per unit privacy cost. As shown in Fig. 13, we generally see strongly diminishing returns past the 2nd feature, with little gain beyond the 3rd or 4th feature for the real-world datasets (little gain beyond the 2nd for the simpler, synthetic cases). This pattern seems to hold broadly across data sets, and is compatible with the convergence of $f^{\prime }$ to f as more basis functions are selected. In practice, it thus seems likely that 2–3 basis functions will be optimal in most settings, though B=1 may be attractive where N is large and privacy preservation is a top priority.

## 5 RELATED WORK

### Federated Learning with Random Features.

The most closely related works in this area are [9, 18, 19, 38]. In [9, 19, 38], random features are used as an approximation method for kernel learning, but not for privacy preservation, which is the focus of our paper. In particular, CodedFedL [38] proposed kernel Fourier feature mapping of the user data in order to tackle a different (the straggler) problem [24, 31], while its potential for privacy-preserving FL as mentioned only as a future direction. None of the prior approaches employ random features in the low-rank regime needed to preserve privacy. [38] solves the specific problem of kernelized linear regression with the Gaussian kernel, while our paper deals with kernel density estimation. [18] proposed FD-SKL – a federated doubly stochastic kernel learning algorithm that utilized random features to approximate the kernel mapping function, assuming vertically partitioned data, and proved that FD-SKL has a sublinear convergence rate. The authors could guarantee data privacy under the semi-honest assumption, but did not quantify the degree of disclosure or consider basis-set restrictions to enhance privacy, and did not analyze privacy-utility tradeoffs. To the best of our knowledge, our paper is the first to explicitly employ projection to small numbers of spatially delocalized random features as a privacy protection mechanism in federated learning, and characterize the privacy-utility tradeoff with federated random feature learning across theory, simulation, and real-world data.

### Privacy-preserving techniques for Federated Learning.

A range of privacy-preserving techniques have been developed and added onto the basic federated learning framework, including differential privacy (DP) on the mobiles and/or the server [27, 34], secure aggregation [7, 25], etc. The state-of-the-art technique for adding carefully calibrating noise [13, 40] is DP, including central DP [45] and local DP. Central DP relies on a trusted curator to add noise centrally [46]. Local DP removes the need for a trusted curator by adding a perturbation to each user’s data (and/or model updates) locally; it provides the strongest privacy guarantees at the expense of loss in utility [2, 28]. Distributed DP with secure aggregation, combines the best of both worlds, and has recently been applied to location heatmaps in [3], which is most closely related to our setting. By introducing a perturbation to the data and/or updates, these noise-adding schemes can provide privacy guarantees against adversaries with arbitrary background knowledge at the cost of decreased learning efficiency. Our key intuition is that, in the spatial setting, the added noise has been traditionally spatially localized. Fig. 1 (lower row) provides an intuition: to add enough noise that an adversary has little idea where a user resides, one may have to remove most of the information content in a user’s signal. As our work shows, this problem can be overcome by using a different privacy-preserving scheme: adding spatially delocalized noise on each device. In summary, this paper introduces a different and orthogonal idea to noise-adding and secure aggregation; it enhances the toolbox of privacy-preserving techniques and can be combined with some of them.

### Location Privacy.

With the increasing need for location-based services (LBS), considerable prior work has evaluated location privacy and compared various privacy-preserving techniques in centralized [12, 27, 36] or federated [4] settings. [8] pointed out that the utility in mobile crowdsource data lies in the measurements, and not in the location itself. They evaluated state-of-the-art location privacy techniques and showed that none is sufficient. In [36], the Dummy-Location Selection (DLS) algorithm was proposed to achieve k-anonymity for users in Location-Based Service (LBS) by carefully selecting dummy locations considering that side information may be exploited by adversaries. However, the anonymization-based mechanism cannot provide a privacy guarantee against attackers with arbitrary background knowledge and differential privacy-based approaches have been applied to LBS to provide strong privacy guarantees [20, 47, 49]. In this paper, we use geo-indistinguishability (GeoInd) [1] as our baseline for comparison. GeoInd is a privacy notion (see Section 4.2.3) based on differential privacy, introduced specifically for location-based systems and shown to be more appropriate than other notions (e.g. local DP) in this context [27].

## 6 CONCLUSION

We have proposed a federated framework to estimate population density that conceals users’ data from a malicious server. Instead of perturbing data or adding spatially local noise, the proposed method projects users’ locations to random spatially delocalized features in Fourier space. We showed that the proposed method has distinct advantages in both density estimation and privacy preservation compared to both Federated KDE and GeoInd DP. Privacy can be protected by using a small number of random features, and we empirically show that when the number of users is large, few random features are still able to achieve good estimation. In addition, we provide theoretical guarantees for privacy-preserving bandwidth selection in the one feature case, which ensures that a target user cannot be localized by any combination of user evaluations sent to the server. Experiments on both synthetic and real-world data empirically show the effectiveness of the proposed method. Our proposed privacy-by-projection technique adds to the privacy-preserving toolbox for federated analytics, and can be used on its own for location data or in combination with other privacy-preserving techniques.

## Figures and Tables

**Figure 12:**
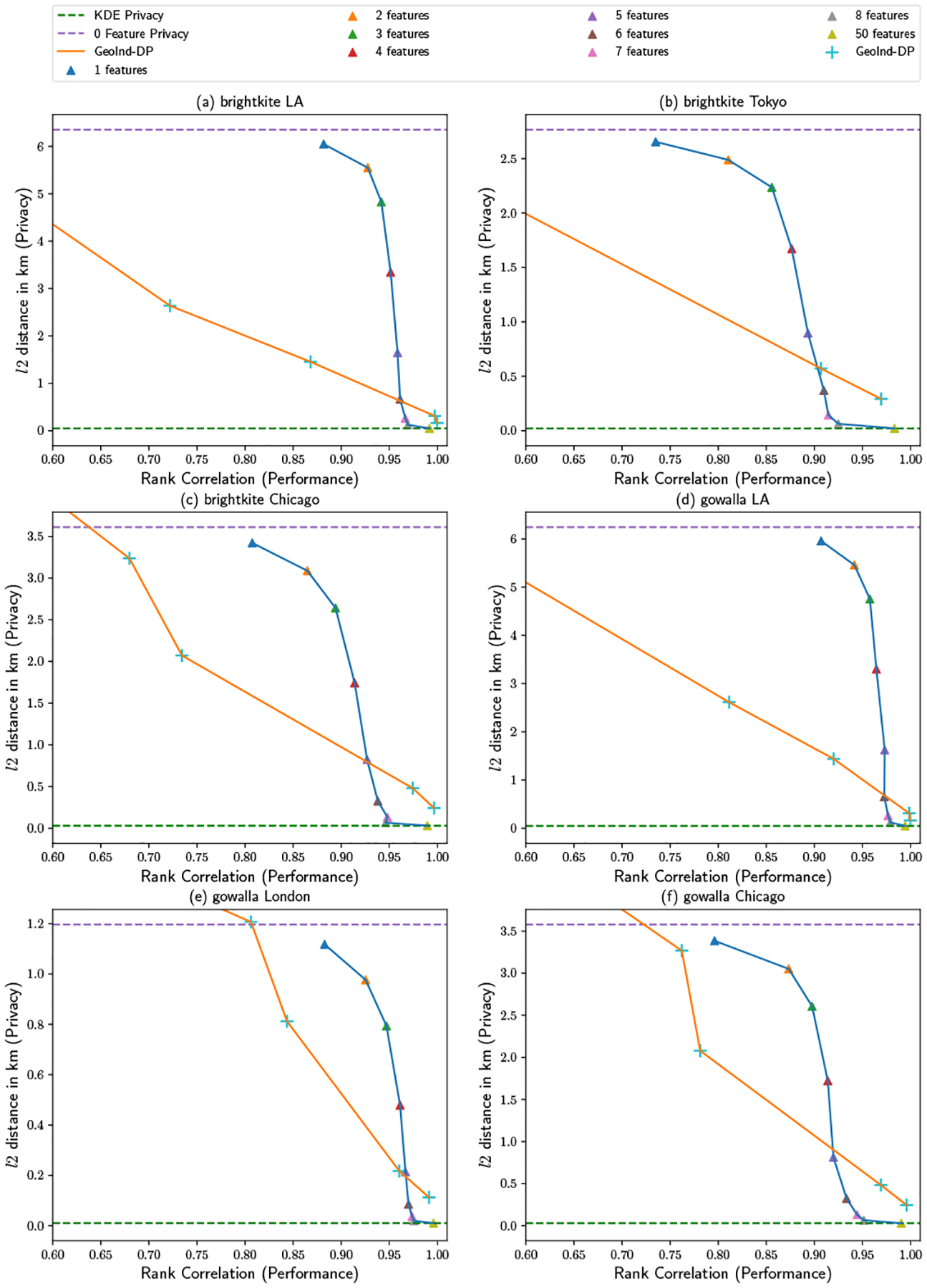
Real-world Performance and Privacy Trade-off

**Figure 14:**
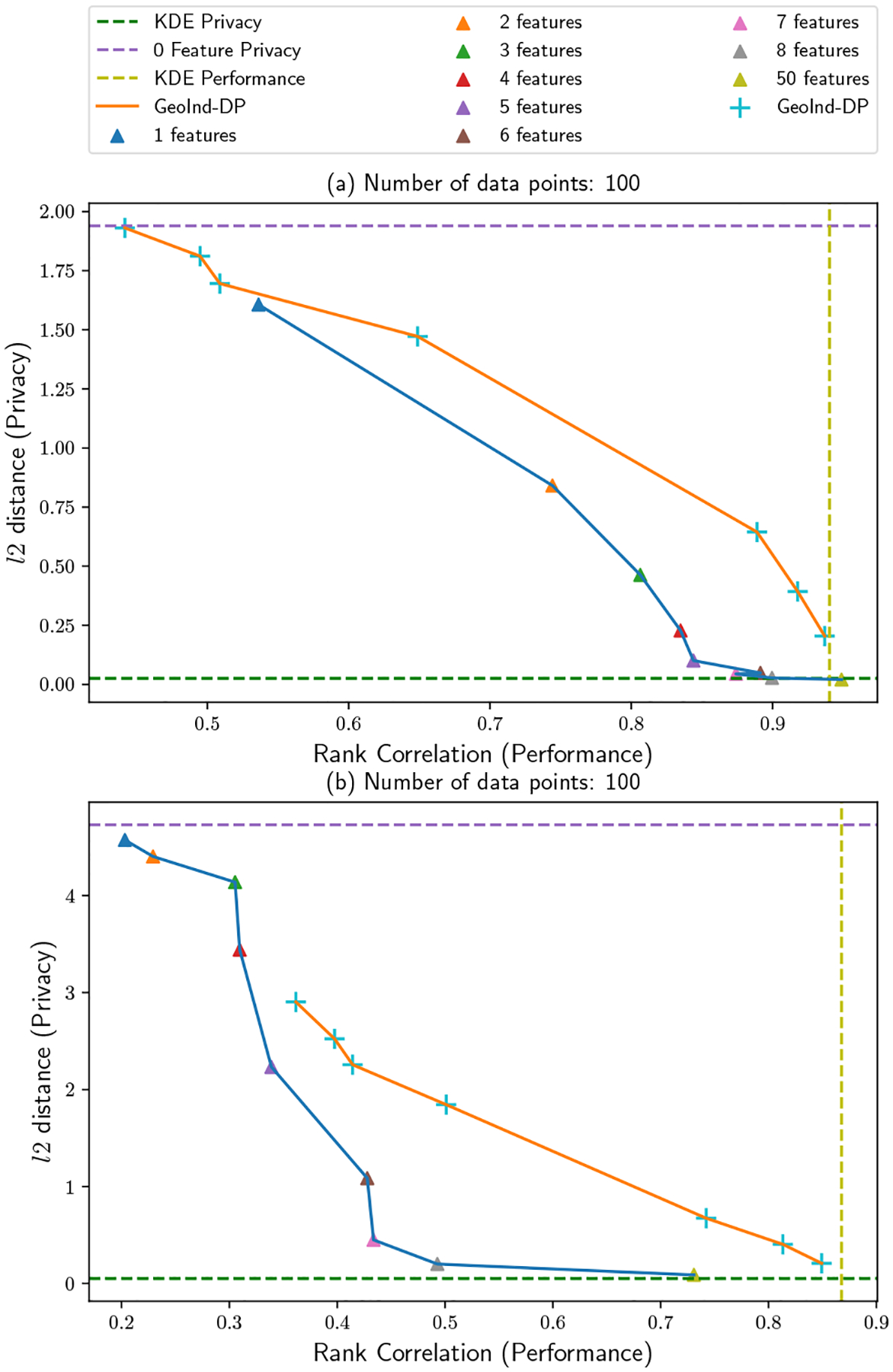
Synthetic datasets with 100 samples

**Table 1:** Real-world data details

	LA	London	Tokyo	Chicago
brightkite	34.11246–118.42099 h=7e−3 #=59307	N/A	35.70064 139.75249 h=1.4e−3 #=20432	41.93045–87.66701 h=3e−3 #=18172
gowalla	34.11246–118.4209 h=7e−3 #=40259	51.52708–0.16971 h=2.2e−3 #=22013	N/A	41.93045–87.66701 h=3e−3 #=28139

## Footnotes

1 “Spatially delocalized function” here refers to a function that is spread out over space, i.e., it spreads the high-probability locations over the entire plane.

2 Data is publicly available at https://snap.stanford.edu/data/

3 Equivalently, the definition can be formulated as $K\left(x\right)\left(Z\right)\leq e^{\epsilon d\left(x,x^{\prime }\right)}K\left(x^{\prime }\right)\left(Z\right)$ for all x, $x^{\prime }\in 𝒳$, Z⊆𝒵. Note that for all points $x^{\prime }$ within a radius r from x, the definition forces the corresponding distributions to be at most ϵr distant. 𝒳 here means points of interest, typically the user’s possible locations and 𝒵 means a set of possible reported values.

## A PROOFS

### A.1 Proof of Lemma 3

Proof. Since all bands are orthogonal to the direction of random vector ω, only consider ${\text{g}}_{mn}$ along the direction of ω, and the period T along this direction is $T=\frac{2\pi }{∥\omega {∥}_{2}}$. To ensure at least j bands appear, we need

(19)
$$jT=\frac{2\pi j}{∥\omega {∥}_{2}}<l$$

since ω is sampled from $𝒩\left(0,h^{-2}\text{I}\right)$ and each dimension of ω is independent from each other, $∥\omega {∥}_{2}$’s distribution is equivalent to the distribution of $\frac{1}{h}\sqrt{Z_{1}^{2}+Z_{2}^{2}}$, where $Z_{i}$ is an independent random variable sampled from normal distribution. Therefore, the sum X of $Z_{1}^{2}$ and $Z_{2}^{2}$ follows chi-squared distribution with 2 degrees of freedom $X\sim \chi^{2}\left(2\right)$. Then, (19) can be rewritten as

(20)
$$\sqrt{X}>\frac{2h\pi j}{l}$$

The probability of (20) holding is

(21)
$$1-P\left(X\leq {\left(\frac{2h\pi j}{l}\right)}^{2}\right)$$

To quantify (21), one can simply leveraging CDFC(⋅) of chi-squared distribution with 2 degrees of freedom. When ${\left(\frac{2h\pi j}{l}\right)}^{2}<\gamma$, the probability in (21) is no less than 1−C(γ). □

### A.2 Proof of Lemma 4

Proof. As stated in Lemma 3, period $T_{0}$ of bandwidth $h_{0}$ is

(22)
$$T_{0}=\frac{2\pi }{∥\omega {∥}_{2}}=\frac{2\pi h_{0}}{\sqrt{Z_{1}^{2}+Z_{2}^{2}}}$$

With a small enough $h_{0}$, there will be a large number of bands across the area. Next, to ensure new bandwidth $h>h_{0}$ has overlapping bands, the period T of bandwidth h should satisfy

(23)
$$\frac{T_{0}}{T}=\frac{\frac{1}{4}}{n+\frac{1}{4}}=\frac{1}{4n+1}$$

T can also be written as the form of (22). Substitute (22) and the similar expression of T into (23), the new bandwidth can only be selected with

$$h=\left(4n+1\right)h_{0}$$

## B MORE ON EXPERIMENTS

### B.1 More Details on the Experimental Setup

In Section 4, we consider four cities: LA, London, Tokyo and Chicago. The areas we are interested to estimate are specified by two pairs of latitude and longitude. In particular, for each selected city, the two pairs, bandwidth selection and number of users inside the areas are:

Two data sets (London for brightkite and Tokyo for gowalla) were substantially smaller than the other datasets (by e.g. an order of magnitude), and in the London case, the majority of data points were in one single location (Trafalgar Square). We do not employ them in the real-world analysis.

### B.2 Additional experiments

We repeated the experiments of the main paper, but with a smaller number of evaluations points. The experimental details are the same as in Fig. 10 and 11. Fig. 14 shows the results, i.e., the privacy-performance tradeoff in synthetic data scenarios (a) and (b) with only N=100 samples.

One can observe that neither privacy-preserving method performs well in that regime, though the DP-based method generally shows a better performance. This is not a surprise since KDE is a non-parametric density estimation method, and is expected to not work well with limited data. Intuitively, bandwidths in small-n settings tend to be large, which reduces the relative performance loss from adding noise in the DP-case (since this is effectively smoothed out). By contrast, this does not compensate for the overall loss of information using the delocalized method. This underscores the results of Fig. 10 and 11, showing an increasing advantage of the projection method versus with data size. When more users join the system, fewer random features need to be used to obtain good resolution and more refined bandwidths become optimal, both of which enhance privacy for the projection technique.
